# Pangenomics insights of *enterococcus faecium* human isolates and identification of novel therapeutic targets by in silico subtractive genomics

**DOI:** 10.1007/s42770-026-01983-z

**Published:** 2026-06-03

**Authors:** Yngrid Victória Cassiano Mascarenhas, Andrei Giacchetto Felice, Felipe Lucas Zen, Victor Augusto Sallum Ceballos, Siomar de Castro Soares

**Affiliations:** 1 Laboratory of Neurobiology, Department of Physiology, Federal University of São Paulo, São Paulo, Brazil; 2 Laboratory of Immunology and Bioinformatics, Department of Microbiology, Immunology and Parasitology, Federal University of Triângulo Mineiro, Uberaba, MG Brazil; 3 Laboratory of Immunology and Bioinformatics, Department of Microbiology, Immunology and Parasitology, Institute of Biological and Natural Sciences, Federal University of Triângulo Mineiro, Uberaba, MG CEP: 38025- 180 Brazil

**Keywords:** *Enterococcus faecium*, Pangenomics, Drug targets

## Abstract

*Enterococcus faecium* is a Gram-positive bacteria that infects the human gastrointestinal tract and it is a leading cause of hospital-acquired infections, due to its ability to cause various types of infections, such as endocarditis, bacteremia, urinary tract infections, and others, exacerbated by its multidrug resistance, notably to vancomycin. Because they are linked to major infections that are difficult to manage, and also due to the widespread acquisition of resistance genes, management remains difficult. This study employs pangenomic analyses and subtractive genomics to explore genetic diversity and identify novel therapeutic targets across 20 human-derived *E. faecium* genomes. Phylogenomic analyses revealed four distinct clades, with genomic rearrangements and horizontal gene transfer events underscoring adaptive evolution. Comparative genomics identified 20 pathogenicity islands and 12 resistance islands, alongside pan-resistome profiling highlighting prevalent resistance to aminoglycosides, elfamycins, and glycopeptides (e.g., vancomycin in 14/20 strains). Core genome analyses, filtered for non-human homologs, prioritized cytoplasmic proteins critical for survival. Subtractive genomics predicted five high-confidence drug targets: phosphocarrier protein HPr (metabolic regulation), GNAT family N-acetyltransferase (antibiotic resistance), translation initiation factor IF-1 (protein synthesis), HU family DNA-binding protein (genome stability), and a sugar-binding domain protein (nutrient uptake). Structural modeling identified these targets as druggable with conserved roles in bacterial viability. This integrative approach elucidates *E. faecium’s* genomic plasticity and resistance mechanisms while proposing candidates for targeted therapies, addressing the urgent need for novel interventions against this resilient pathogen.

**Supplementary Information:**

The online version contains supplementary material available at 10.1007/s42770-026-01983-z.

## Introduction

### *Enterococcus* and *enterococcus faecium* genus

The *Enterococcus* genus is a group of Gram-positive bacteria belonging to the *Enterococcaceae* family, which can be alpha-hemolytic or non-hemolytic, and may present themselves in pairs or short chains. This genus includes several species, with *Enterococcus faecalis* and *Enterococcus faecium* being the most common and clinically relevant. The gastrointestinal tract is the main human reservoir for Enterococcus; although they are also found in the oral cavity, gallbladder, urethra and vagina, less frequently. In feces of healthy adults, it is common to find high concentrations of *E. faecalis*, 90 to 95% of the species are found, while E. faecium is detected in lower concentrations, ranging from 5 to 10% [1].

Bacteria belonging to the *Enterococcus* genus were initially classified as Gram-positive enteric cocci and, with the implementation of the Lancefield serological screening system in the 1930 s, they were included in the genus *Streptococcus* of group D. Later, in 1984, the classification of these species, now in the genus *Enterococcus*, was established based on DNA-rRNA and 16 S rRNA homology studies, which revealed significant differences between *Streptococcus* and *Enterococcus*, leading to the reclassification of these microorganisms [2]. Since then, two distinct species of this genus, *E. faecalis* and *E. faecium*, have gained greater notoriety as some of the most frequently found nosocomial pathogens in healthcare settings worldwide [3]. Currently, *E. faecium*, for example, corresponds to the third largest cause of hospital infection, in addition to emerging as an important opportunistic pathogen that causes infections in the community, especially in immunocompromised individuals or with associated comorbidities [4]. In hospital settings, this genus can cause medical device-related infections, such as catheter-associated urinary tract infections or intravenous catheter-related bloodstream infections. They may also be responsible for respiratory tract infections, surgical wound infections, and other nosocomial infections. In the community, the infection caused by this pathogen is more related to people with compromised health conditions, and can lead to urinary tract infections, skin and soft tissue infections, and intra-abdominal infections.

*Enterococci* are linked to a considerable number of difficult-to-manage infections, due to the acquisition of genes that confer resistance to current treatments, which results in the deterioration of the patient’s condition and, consequently, in the worsening of the clinical condition [5]. In recent decades, there has been a significant increase in the rates of acquired antimicrobial resistance in *E. faecium*, including resistance to vancomycin, an antibiotic of critical importance for the treatment of severe bacterial infections [6]. Serious infections caused by vancomycin-resistant *E. faecium* (VRE) have consistently presented clinical challenges, requiring combined therapeutic approaches and management of treatment-associated toxicity. Despite the introduction of new antibiotics with efficacy against VRE in the therapeutic arsenal, significant challenges remain. An understanding of the drivers of resistance development in VRE, the dynamics of gastrointestinal colonization and resistance to microbiota-mediated colonization, as well as the mechanisms of resistance to currently available therapies, will allow healthcare professionals to be better prepared to address these challenges associated with hospital-acquired pathogens [7].

To achieve effective prevention and reduction of nosocomial infections caused by VRE, it is crucial to also direct efforts to decrease the transport and spread of VRE. Effective control of *E. faecium* and other resistant bacteria requires the implementation of appropriate infection prevention and control measures, such as hand hygiene, proper hygiene practices, isolation of infected patients, prudent use of antibiotics, and education of healthcare providers on the appropriate use of antimicrobials [8]. In addition, continued research and development of new antimicrobials and alternative therapies, along with efforts to improve awareness of antimicrobial resistance, are essential to combat the spread of resistant *E. faecium* and improve the treatment of these infections.

In this context, it is important to analyze the pan-genomics of this species, an area introduced by Tettelin et al. in 2005 [9], which presents a more comprehensive view of the genome of a species or genus. This approach involves the comparative analyses of multiple genomes of different isolates of the same species in order to identify the complete and non-redundant repertoire of genes present in the compared genomes. The availability of a large number of genomes of different isolates of the same pathogen has provided the opportunity to investigate genomic characteristics intrinsic to the species [10]. In this way, subtractive genomics becomes an interesting area to analyze these genes found in pan-genomics and identify those responsible for survival mechanisms that may become possible new drug targets [11].

## Materials and methods

### Pan-genomic analysis

#### Genome selection

In order to perform pan-genomic analyses with strains isolated from the human host, the genomes of different strains of the species *E. faecium* that are deposited in a public database, the *National Center for Biotechnology Information* (NCBI - https://www.ncbi.nlm.nih.gov/*)*, were used. Since there are more than 30 thousand genomes deposited in this database, we initially filtered to those that were annotated and are present in the Refseq database and excluded atypical genomes, leaving 4083 genomes. This step was also necessary to warrant the quality of the data and homogeneity of the annotation. Also due to computational and software limitations, it was necessary to reduce the dataset even more; then, we used the phylogenetic tree provided by the NCBI taxonomy to select 20 genomes isolated from human hosts that would be representative of the clades found. Some information on these genomes is available in Table 1.

In addition, 1 genome of the species *Enterococcus casseliflavus EC20* (GCF_000157355.2) was chosen as an outgroup for phylogenomic analyses. In order to perform the prediction of the pathogenicity islands (PAIs) and resistance islands (RIs), another outgroup was used, *Enterococcus gallinarum* FDAARGOS-163 (GCF_001558875.2), totaling 24 genomes in the analyses. These species used as outgroups are not commonly pathogenic, but the literature mentions that they may be indirectly associated with some pathology, for this reason it was decided to use two species commonly known to be non-pathogenic in healthy individuals (opportunistic pathogens) to reduce the odds of false negative predictions of PAIs. These genomes were obtained through the ftp protocol, in faa and fna formats, using *in-house scripts*. All genomes used in this work were downloaded in December, 20, 2023.

**Table 1:** *Enterococcus faecium* genome information

Organism/ Name	Strain	Size (Mb)	GC (%)	Genes	Proteins	Geographical location	Host	Assembly ID
***Enterococcus faecium***	E8377	3.253	37.5	3.237	3.049	Netherlands	Human hospitalized patient	*GCF_900639595.1*
***Enterococcus faecium***	Aus0004	3.020	38	3.118	2.919	Australia	Human	*GCF_000250945.1*
***Enterococcus faecium***	E745	3.168	37.5	3.149	2.943	Netherlands	Human hospitalized patient	*GCF_001750885.1*
***Enterococcus faecium***	DO	3.053	37.5	3.007	2.779	United States of América	Human	*GCF_000174395.2*
***Enterococcus faecium***	E6055	3.040	37.5	2.996	2.798	-	-	*GCF_900639345.1*
***Enterococcus faecium***	E8423	2.998	37.5	2.959	2.754	Netherlands	Human	*GCF_900639725.1*
***Enterococcus faecium***	Aus0085	3.239	37.5	3.271	2.960	-	-	*GCF_000444405.1*
***Enterococcus faecium***	AALTL	3.112	37.5	3.058	2.854	United States of América	Human	*GCF_002880635.1*
***Enterococcus faecium***	Efaecium_ ER04526.5 A	3.126	37.5	3.087	2.882	United States of América	Human	*GCF_002848725.1*
***Enterococcus faecium***	E243	3.141	37.5	3.111	2.905	United States of América	Human	*GCF_002761275.1*
***Enterococcus faecium***	Dallas 131_2	3.186	37.5	3.117	2.921	United States of América	Human	*GCF_016415465.1*
***Enterococcus faecium***	17–508	2.874	38	2.849	2.644	France	Human	*GCF_018517045.1*
***Enterococcus faecium***	NCTC7174	2.450	38	2.364	2.176	-	-	*GCF_900637035.1*
***Enterococcus faecium***	NRRL B-2354	2.850	37.5	2.773	2.615	-	Milk and dairy utensils	*GCF_000336405.1*
***Enterococcus faecium***	EFE10021	2.615	38	2.495	2.368	-	Human	*GCF_900066025.1*
***Enterococcus faecium***	VB6521	3.248	37.5	3.193	3.009	India	Human	*GCF_017898005.1*
***Enterococcus faecium***	VRE1	3.098	37.5	3.053	2.860	China	Human	*GCF_006007925.1*
***Enterococcus faecium***	SRR24	2.919	37.5	2.887	2.714	China	Human hospitalized patient	*GCF_009734005.1*
***Enterococcus faecium***	A7214	3.109	37.5	3.080	2.866	India	Human	*GCF_012933165.2*
***Enterococcus faecium***	T110	2.738	38	2.647	2.515	-	-	*GCF_000737555.1*

#### Phylogenomic and phylogenetic analysis

The phylogenomic analyses were carried out considering the Gegenees software [12] strategy, in which, through BLAST (Basic Local Alignment Search Tool), it produces a distance matrix based on the percentage of similarity between the genomic fragments of the analyzed species. The 21 genomes (20 *E. faecium* + 1 *E. casseliflavus* lineages) were deposited for analyses in this software, using the custom method of comparison, with 200 bp for the fragment size, 100 bp for the step size and BLASTn; and, the result was plotted on a heat map. The resulting distance matrix could be exported in nexus format to be used in the SplitsTree4 software [13], which aimed to infer the phylogenetic tree. For this, phylogenetic inference was used by the *Neighbor-Joining* method.

#### Average nucleotide identity

Given the results of the previous software, we carried out a more in-depth analysis of the *E. faecium T110* lineage, due to the atypical behavior found. For this purpose, the Average Nucleotide Identity Analysis (ANI calculator) program from the Kostas lab website (http://enve-omics.ce.gatech.edu/ani/index*)* was used, which serves as a widely used measure in comparative genomics to assess the similarity between two genomes at the nucleotide level [14]. The program was used to perform two comparisons against the *T110* lineage, the first against the reference species of the genus of interest, the *E. faecium* SRR24 lineage; and the second against the *Streptococcus pyogenes* lineage NCTC12064 (GCF_900475035.1). The program was used in its default configurations, and for the alignment options, 700 bp was used as the minimum size, 70% as the minimum identity and 50 as the minimum alignment; and, for fragment options, 1000 bp for window size and 200 bp as step size.

#### Gene synteny

For the gene synteny analysis, the Mauve software [15] was used, where 2 groups of analyses were performed, the first one using all 21 genomes (20 *E. faecium* + 1 *E. casseliflavus*) and a second one using only the clusters found in the SplitsTree4 software. Mauve was used to identify genomic rearrangements, inversions, duplications, and deletions, in addition to facilitating the visualization of similarities and differences between the genomes analyzed. The program was used in its default settings, being: the matrix resulting by the HOXD method, gap open score of −400, gap extend score of −30 and a match seed weight of 15.

#### Genome plasticity analysis and genomic island prediction

Genomic Island Prediction Software (GIPSy) is a software developed for the identification and analysis of genomic islands in DNA sequences, which are segments often associated with horizontal gene transfer [16]. These segments have a GC content different from the organism analyzed, a deviation in the codon usage, have the presence of transposases and are usually larger than 6 kb. These regions can be further classified as presence of antibiotic resistance genes, and PAIs, with high concentrations of virulence factors. Two analyses were carried out with 2 opportunistic outgroups, being: *E. faecium* SRR24 x *E. casseliflavus EC20*; and, *E. faecium* SRR24 x *E. gallinarum FDAARGOS-163*.

The results from GIPSy were plotted using the BLAST Ring Image Generator (BRIG), a bioinformatics visualization software that enables the comparative analyses of genomes, creating images of rings that represent the genetic similarity between different sequences identified through the use of BLAST algorithm. In this analysis, the central genome for comparison was the reference genome of *E. faecium*, pre-established by the NCBI (*E. faecium SRR24*), with all formed rings being compared to this central sequence [17]. The same 21 genomes were used in this analysis and the program was used with its default settings.

#### Pan resistome prediction

For the prediction of antibiotic resistance genes (ARGs), the Pan Resistome Analysis Pipeline (PRAP) was used, a platform-independent tool developed in Python 3, which is based on the Comprehensive Antibiotic Resistance Database (CARD) and ResFinder databases. This software enables the identification of resistance-related genes in whole genomes [18]. In addition, the generated annotations are used in the characterization of the pan-resistomes, which makes it possible to analyze the distribution of ARGs among the genomes analyzed. For pan-resistome analyses, the CARD database was used exclusively, as it offers a comprehensive catalog of ARGs. The program was used with the -m N1 option, to select the CARD database, and following the rest of the default settings, running locally.

### Reverse vaccinology and subtractive genomics

Orthofinder software [19] was used to carry out the search for orthologous proteins among the *E. faecium* strains. Through the Diamond and MCL algorithm, the program identifies homology regions between the genes of the species and separates these groups of orthologous proteins. After separation, *in-house* scripts were applied on the orthofinder result to separate these proteins into the pangenome subgroups: core genome (proteins present in all lineages), accessory genome (proteins present in more than one, but not all lineages), and singletons (present in only one lineage).

In the core genome there are usually proteins that are involved in important mechanisms for the survival of the microorganism [11], so it was chosen to continue the analyses. As a way to avoid possible adverse reactions, the core genome was compared with the human proteome through the NCBI’s BLASTp tool [20] and then only the proteins of the microorganisms that did not have homology in this analysis were selected for the next step.

#### Prediction of subcellular localization of proteins

Protein subcellular localization prediction was performed using SurfG+ software [21], which allows for the identification of specific locations of proteins within the cell based on their sequences. The software separates proteins into membrane, secreted or PSE (typically used for vaccine candidate research), and cytoplasmic (typically used for drug target research). Proteins categorized as cytoplasmic were then targeted for detailed analysis to identify potential drug targets, given that they are often associated with metabolic pathways critical to the organism’s survival. This approach allows a deeper understanding of the functions of these proteins and their implications for therapeutic strategies.

#### Selection of potential drug targets

For drug target prediction, the MHOlline software was used, which constitutes a pipeline designed for the analysis of protein structures in structural genomics projects [22]. This software integrates a variety of specific programs and methods that allow performing analyses such as comparative modeling, structural quality (ranging from very high to very low), and stereochemical quality alignment (Smith & Doe, 2021; Zhang, 2020). The final modeling was separated into G0, G1, G2 and G3, as follows: G0: non-aligned sequence; G1: E-value > 10 × 10-5 or identity < 15%; G2: E-value 10 × 10-5, identity ≥ 25% and LVI (Length Variation Index) ≤ 0.7; G3: E-value ≤ 10 × 10-5, identity ≤ 15% to < 25% or LVI > 0.7. In the result, proteins with ‘very high’, ‘high’, and/or ‘medium to good’ quality can be considered good candidates and are within the G2 quality group.

#### Functional annotation of proteins

The final candidates were characterized using different software and databases, such as UniProt [23], NCBI, MHOLline, SurfG+. Table 2 summarizes this information.

### Docking analyses

Molecular docking analysis is a widely used computational method to investigate molecular interactions and to support drug discovery or drug repositioning [24]. For this approach, the 3D structures of each identified protein, generated using the MHOLline server, were evaluated for structural quality. The Ramachandran plots [25] for each protein are presented in Figure S1. Subsequently, the structures were prepared using AutoDock Tools [26] to assign charges to the protein targets and to set up the docking grid boxes.

DogSiteScorer, a Proteins Plus tool [27], was used to identify potential binding pockets within each 3D structure. A ligand library containing 5,008 drug-like molecules (i.e., natural products) was prepared according to the methodology described by Vilela-Rodrigues et al. [28] and used as input for molecular docking. AutoDock Vina [29] was employed to perform the docking simulations between the protein targets and each ligand. An in-house script was used to select the top 10 compounds based on binding affinity, and UCSF Chimera [30] was used to visualize and generate 3D representations of the docking interactions.

## Results and discussion

### The distance matrix reflects a high similarity between the lineages

**Fig. 1:**
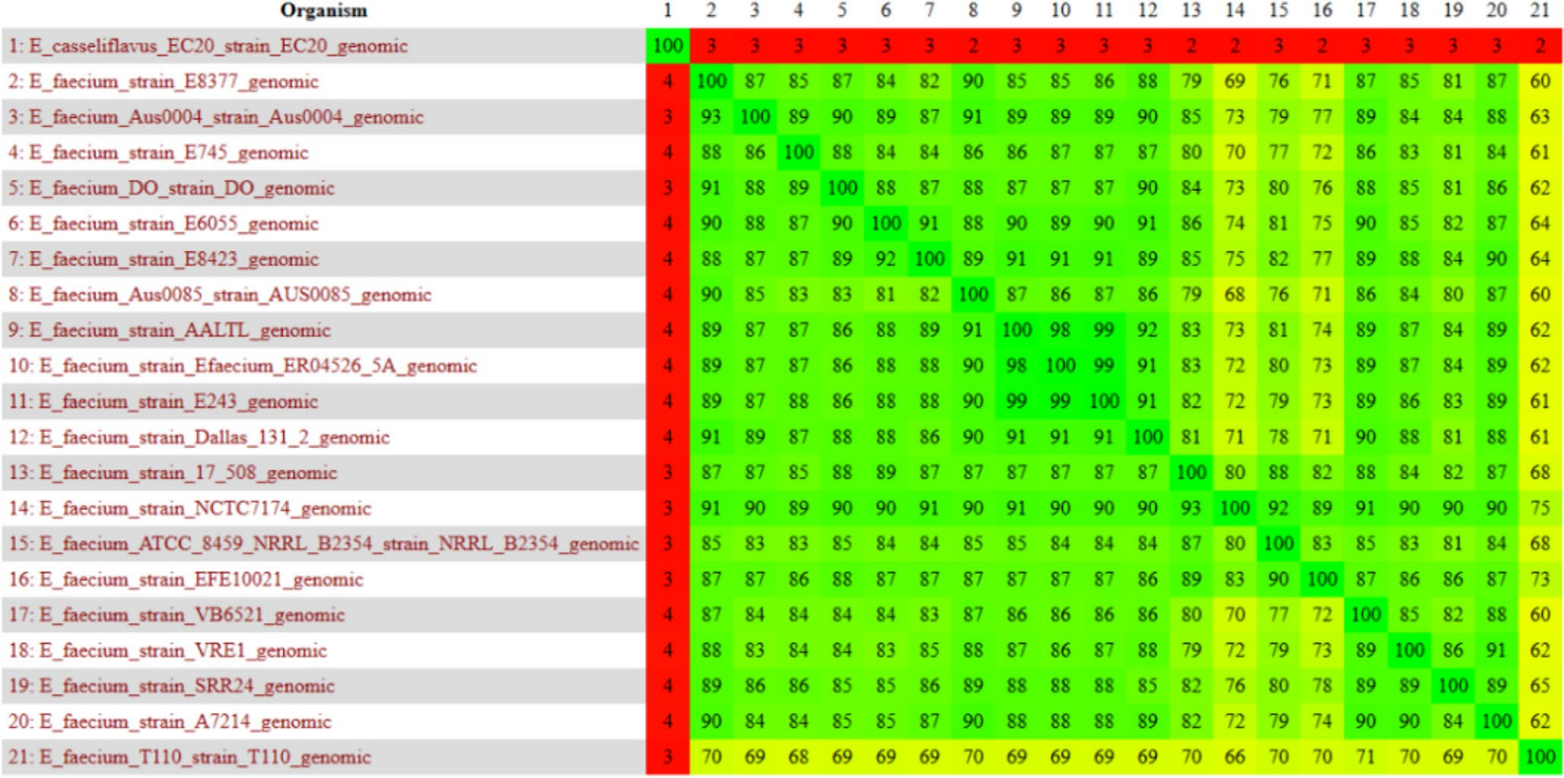
Similarity heatmap generated with Gegenees software. Red color represents alow degree of similarity; and, green/greenish color a high degree of similarity

The heat map generated by the Gegenees software presents a distance matrix that reflects the similarity between genomes, ranging from 0% to 100%. In the context of this project, the heat map demonstrates a similarity range that oscillates between 60% and 100% (Fig. 1).

Interestingly, when analyzing comparisons between strains of the same species (*E. faecium*), some, such as those corresponding to numbers 14, 15, 16 and 21 (*E. faecium* strain NCTC7174; *E. faecium* strain ATCC 8459; *E. faecium* strain EFE10021; and *E. faecium* T110, respectively), exhibit a lower similarity, ranging from 60% to 83%, when contrasted with the other intraspecies comparisons. In addition to this observation, the *E. faecium* T110 strain presents the lowest similarity, even within this small clade. It is also worth noting that the addition of a species as an outgroup, *E. casseliflavus* EC20, obtained a variation of 2 to 4% of similarity, which was already expected since it is different from the others in the group.

Given this somewhat reduced similarity in this small group, we can raise some hypotheses, such as genetic variation between lineages. These lineages may have undergone mutations or specific genomic rearrangements (Single Nucleotide Polymorphism - SNP) that differentiate them from the others, resulting in discrepancies in gene sequences and, consequently, in their similarity [31]. In addition, the adaptability of specific lineages to different environments or selective conditions can lead to changes in genes that are not present or that become divergent in other lineages [32]. This adaptability may include the acquisition of antibiotic-resistance genes or the loss of non-essential genes, impacting genetic similarity.

Another relevant factor is the effect of natural selection, which may influence the divergence of lineages over time. Lineages that inhabit distinct ecological niches may have evolved to present different genetic characteristics, leading to less similarity between them [33]. Additionally, horizontal gene transfer (HGT) between bacteria may play a significant role. HGT allows lineages to acquire new functions or characteristics, and if some lineages acquired genes through this mechanism, this could explain the variation in similarity compared to other lineages [34].

Finally, limitations in the databases used for the analysis may also influence the results. If less similar strains contain genes that are not well represented in reference databases, this may result in a lower similarity score.

**Fig. 2:**
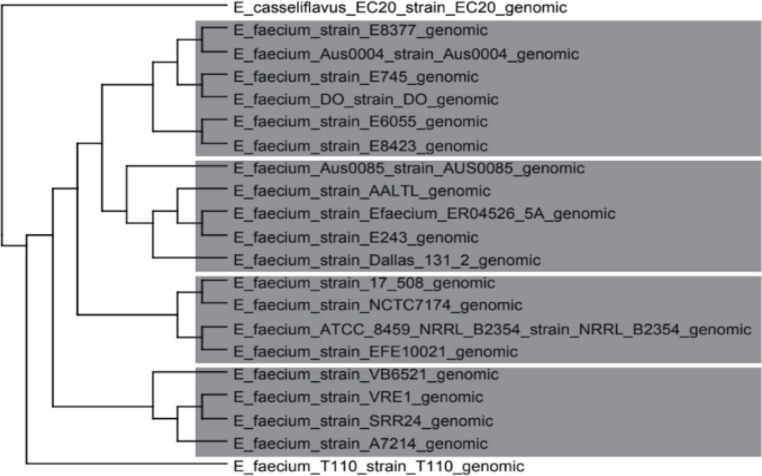
Taxonomic analysis of *Enterococcus faecium* species generated with SplisTree4 software. The branches represent a greater proximity between the species (fraction corresponding to the aligned positions). Formation of 4 clades demarcated by a gray rectangle. The outgroup is rooting the tree (*E. casseliflavus* EC20)

In the phylogenetic analysis, the tree constructed by the SplitsTree4 software shows the ancestral relationships between lineages of the same species when compared with the outgroup of the species *E. casseliflavus* EC20 (Fig. 2). SplitsTree4 uses phylogenetic networks instead of traditional trees and represents the evolutionary relationships between different lineages. This type of representation is especially useful when there are conflicting signals in the data, as in the result previously analyzed (Gegenees), which can occur in recombination events, hybridization, or horizontal genetic exchanges, where it is possible to visualize these inconsistencies in a reticulated structure by this second analysis.

There is the formation of four main clades with high genetic similarity between strains of the same clade compared to strains of distinct clades, which then suggests a genetic proximity. The results follow the same line found in the previous result, and even the same group found previously, and which had a slightly lower similarity than the others (*E. faecium* strain NCTC7174; *E. faecium* strain ATCC 8459; *E. faecium* strain EFE10021) was also formed in a separate clade. This information from these clades follows the same explanation we had previously described, due to the large genetic changes/adaptations that may have occurred throughout evolution [35].

Another result observed through the use of the SplitsTree4 software is the exclusion of the *E. casseliflavus* EC20 and *E. faecium* T110 strains. In the first case, it was expected that *E. casseliflavus* EC20 would not be part of any clade, as it was placed as an outgroup [36]. The same serves to distinguish ancestral characteristics from characteristics derived from a group of organisms under study, known as ingroup, in this case the lineages of *E. faecium*. In the case of the *E. faecium* T110 lineage, it was something atypical deserving to be better analyzed, for this reason we chose to use the ANI program and deepen the analysis.

### The average nucleotide identity corroborates the findings

**Fig. 3:**
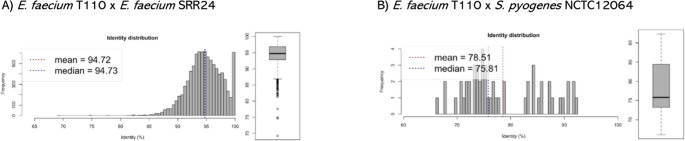
Average Nucleotide Identity of *Enterococcus faecium *T110. In A, the percentage of similarity between the species *E faecium* T110 and *E faecium* SRR24; and, in B, the percentage of similarity between the species *E faecium* T110 and *Streptococcus pyogenes* NCTC12064

Using the ANI program, it was possible to observe in the analysis the percentage of similarity between the *E. faecium* T110 and *E. faecium* SRR24 strains, which resulted in 94.72% (Fig. 3A). And for the percentage of similarity between the *E. faecium* T110 and *Streptococcus pyogenes* lineage, NCTC12064 which resulted in 78.51% (Fig. 3B). The results obtained by the ANI program highlight significant differences in the evolutionary proximity between the analyzed lineages. The 94.72% similarity between *E. faecium* T110 and *E. faecium* SRR24 indicates a close phylogenetic relationship, possibly between lineages of the same species or subspecies that share a high percentage of homologous genes. On the other hand, the similarity of only 78.51% between *E. faecium* T110 and *S. pyogenes* NCTC12064 suggests a more distant phylogenetic relationship, reflecting that they are distinct species with considerable genomic differences [37]. This lower percentage indicates that these lineages have a reduced number of genes in common, which is expected among different bacterial genera.

These results also allow discussions about the degree of genetic variation between lineages of the same species compared to different species and may have practical implications, such as in the identification of virulence factors, antibiotic resistance, or pathogenic potential traits shared by genetically closer lineages [38]. Data in the literature supports these arguments above. In 1990, Murray [2] described that biochemical and genetic analyses caused a group of species belonging to the genus *Streptococcus* to change their nomenclature to *Enterococcus* in 1984. An ANI of 95% corresponds to a similarity of 70% in DNA-DNA hybridization, being used to distinguish between different species [14], so this result may suggest the reason why there was a change in the nomenclature of this species.

### Despite their similarities, several translocation, deletion, inversion, and duplication events have occurred

**Fig. 4:**
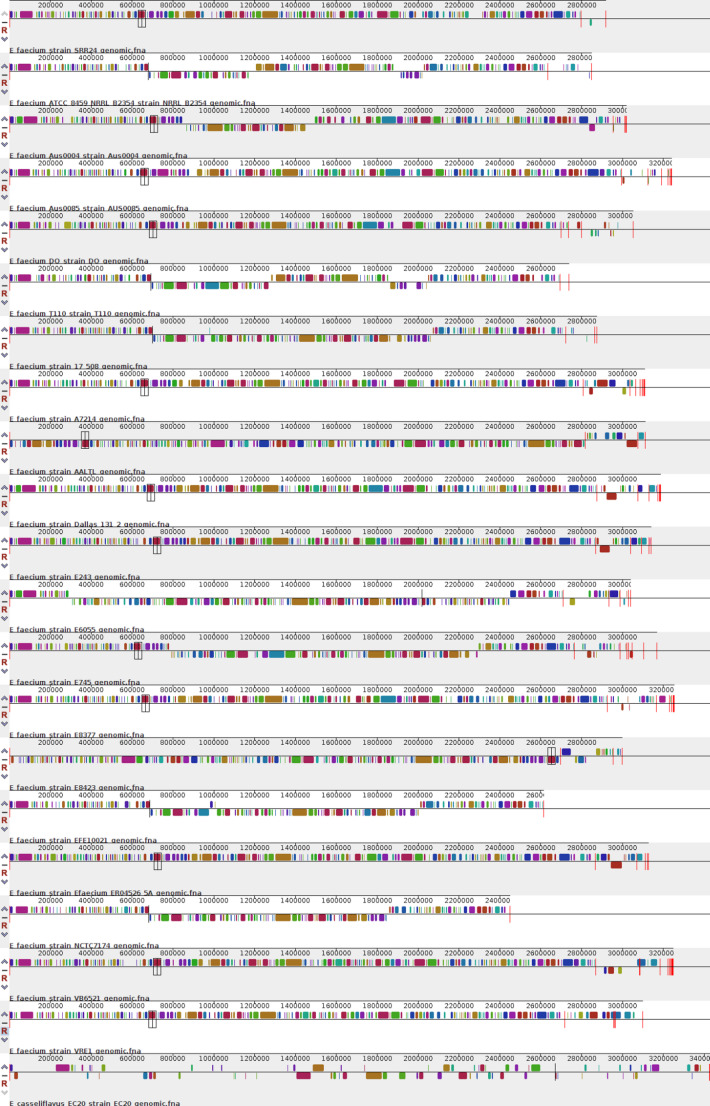
Gene synteny analyses of all 20 genomes from *E. faecium*. Each color bar represents blocks of neighbouring genes shared between each genome, called collinear regions. Each line plot represents each of the strains present in the work. The first line represents the reference strain used in the work

**Fig. 5:**
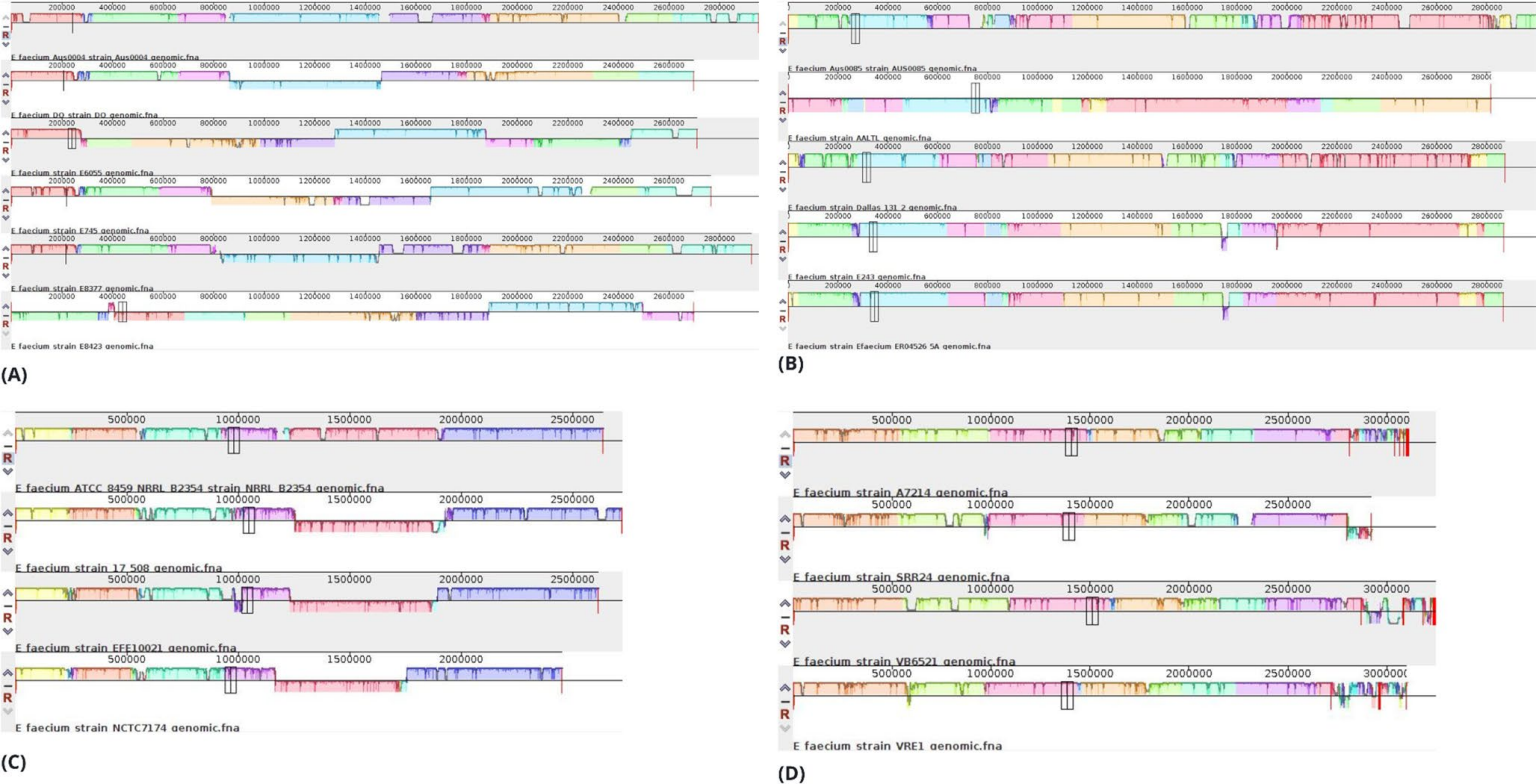
Gene synteny analysis based on clades found in the taxonomy. Construction of multiple genome alignments performed by the Mauve software on clades previously found by the SplitsTree4 analysis. (**A**) represents the 1 st clade (*E. faecium* Aus0004; *E. faecium* DO; *E. faecium* E6055; *E. faecium* E745; *E. faecium* E8377 and *E. faecium* E8477); (**B**) the 2nd clade (*E. faecium* Aus0085; *E. faecium* AALTL; *E. faecium* Dallas 131-2; *E. faecium* E243; *E. faecium* ER04526_5A); (**C**) the 3rd clade (*E. faecium* ATCC 8459 NRRL-B2354; *E. faecium* 17–508; *E. faecium* EFE10021; *E. faecium* NCTC7174); and (**D**) the 4th clade (*E. faecium* A7214; *E. faecium* SRR24; *E. faecium* VB6521; *E. faecium* VRE1)

Using the MAUVE software, the first analysis of all the 20 genomes of *E. faecium* studied here (Fig. 4) revealed a set of genomic rearrangements, including translocations, deletions, and inversions, which reflect the evolutionary process of the species over time. This evolutionary dynamic is further corroborated by subsequent analyses (Fig. 5), performed with the clades identified in SplitsTree4, offering a complementary and detailed view of the phylogenetic relationships between the genomes.

The clades resulting from the SplitsTree4 software are organized as follows: 1 st clade containing *E. faecium Aus0004*, *E. faecium DO*, *E. faecium E6055*, *E. faecium E745*, *E. faecium E8377* and *E. faecium E8477*; 2nd clade with *E. faecium Aus0085*, *E. faecium AALTL*, *E. faecium Dallas 131-2*, *E. faecium E243* and *E. faecium ER04526_5A;* 3rd clade composed of *E. faecium ATCC 8459 NRRL-B2354*, *E. faecium 17–508*,* E. faecium EFE10021 and E. faecium NCTC7174;* and 4rth clade harboring *E. faecium A7214*, *E. faecium SRR24*, *E. faecium VB6521* and *E. faecium VRE1.*

In Fig. 4, there are very small and highly fragmented blocks, suggesting the strains have undergone many evolutionary events. In this case, the ones that differ the most are the *E. faecium* E8423 and *E. faecium* AALTL lineages, which underwent an inversion process in almost the entire genome, approximately 2.8 Mb in comparison to the reference genome. The other genomes presented translocation events of small portions of genes. In Fig. 5, in general, all clusters present larger block sizes and fewer evolutionary processes, corroborating with the previous steps that these species are closer/more clonal. The 1 st clade (Fig. 5A) and the 2nd clade (Fig. 5B) were the ones that suffered the most from these processes, with some inversions and translocations, compared to the 3rd clade (Fig. 5C) which had deletions and only one inversion in the pink block; and to the 4th clade (Fig. 5D) that had deletions and a few translocations.

The identification of genomic rearrangements, such as inversions and translocations, allows us to explore how these events contributed to the diversification of lineages over time, impacting the adaptation and survival of variants in different environments. Furthermore, the greater similarity between variants of the same clade, compared to the similarity between distinct lineages, suggests a stronger evolutionary proximity within clades, possibly indicating that these lineages evolved from a more recent common ancestor. This finding opens the possibility of discussing how distinct selective forces acted on each group over time. The presence of conserved regions between genomes, in turn, highlights the functional importance of these regions for the viability of lineages. Analysis of the functions associated with these regions can reveal which genes are essential and which present adaptive variations, offering clues about the biology and specific adaptations of lineages. Finally, depending on the organism studied, the analysis of conserved regions and genomic rearrangements can also provide valuable information on antibiotic resistance or other environmental factors, which is particularly relevant for strains associated with pathogenic traits or hospital environments, where genomic adaptation is essential for persistence [39].

### Genome plasticity, genomic islands and horizontal gene transfer

**Fig. 6:**
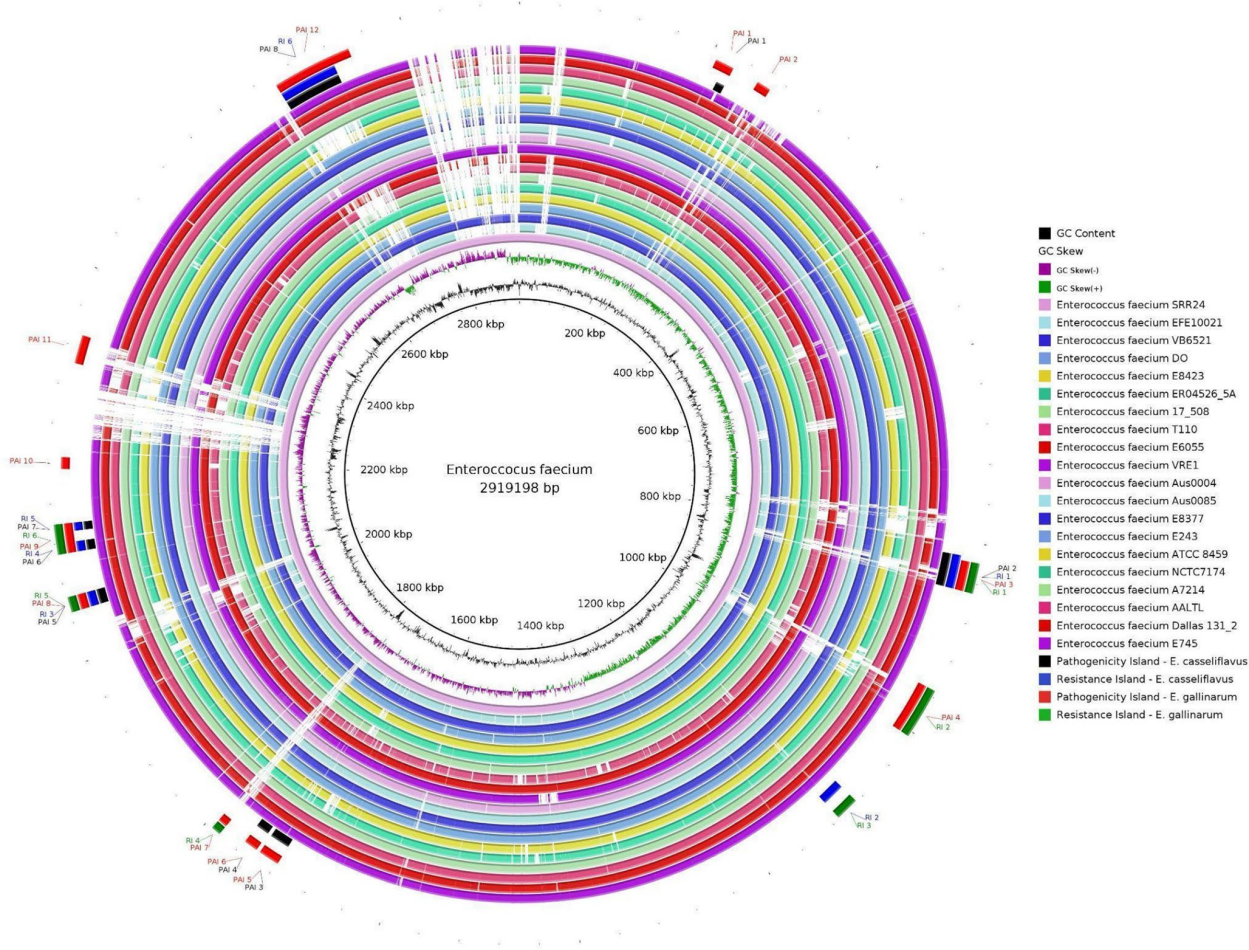
Genomic plasticity of *Enterococcus faecium* strains. Rings created by the BRIG software. Each of the colored rings represents a strain used in the work. White regions on the map represent gene deletion zones compared to the reference genome, *E. faecium* SRR24. The four outermost rings represent the genomic islands

Each of the rings is representative of the strains used in the study and we can therefore observe a high clonality between the strains, with some deletion regions (in white). The results of the GIPSy analysis were obtained using two opportunistic outgroups: *E. faecium* SRR24 x *E. casseliflavus* EC20, which presented 8 PAIs and 6 RIs, and *E. faecium* SRR24 x *E. gallinarum* FDAARGOS-163, with 12 PAIs and 6 RIs. In total, 20 PAIs and 12 RIs were identified among the genomes analyzed (Fig. 6).

When analyzing the circular map image, a very homogeneous group of organisms is found, sharing many gene regions, though some deletion regions (blank spaces on the map) can be found. The detection of deletion regions by using BRIG can be important for many reasons, such as analyzing the loss of adaptive genes that can signal evolution and adaptation to different environments. These regions can contribute to unique characteristics, such as antibiotic resistance or interactions with hosts, and in genetic variants, they can serve as markers to differentiate genetic lineages [17].

Lineages with distinct genetic content (such as the presence of genomic islands) may be due to horizontal acquisition of mobile genetic elements or gene duplication events. Through comparative genomics, a probable evolutionary trajectory of *Enterococci* has been outlined, and from the literature, it is possible to demonstrate that both *E. faecalis* and *E. faecium* arose independently, largely due to gene acquisitions via mobile elements. *Enterococci* have a coevolution with their hosts that extends over hundreds of millions of years and, as shown by the result of the Mauve software, there is an evolution between the *E. faecium* lineages.

The 20 PAIs contain virulence genes that include, among others, those related to host adhesion, a crucial factor for colonization and invasion of target tissues. On the other hand, the 11 RIs reveal genes associated with antibiotic resistance, which highlights an adaptation of the strains to environments with antimicrobial selective pressure (Table S1). There are suggestions that hospital *Enterococci* have an enhanced ability to cope with extreme conditions compared to other members of the genus, which have maintained an ancestral state [40]. This concept is corroborated by the fact that *E. faecium*, in particular, exhibits a remarkable phenotype of tolerance to nutrient deprivation, indicating specific adaptations in this species that contribute to its environmental persistence in healthcare settings [3]. Thus, this genomic profile may indicate an increased risk to public health, highlighting the need for constant surveillance and new approaches to control and treat infections associated with these genetically diverse and adaptable lineages.

### Important genes were founded

**Fig. 7:**
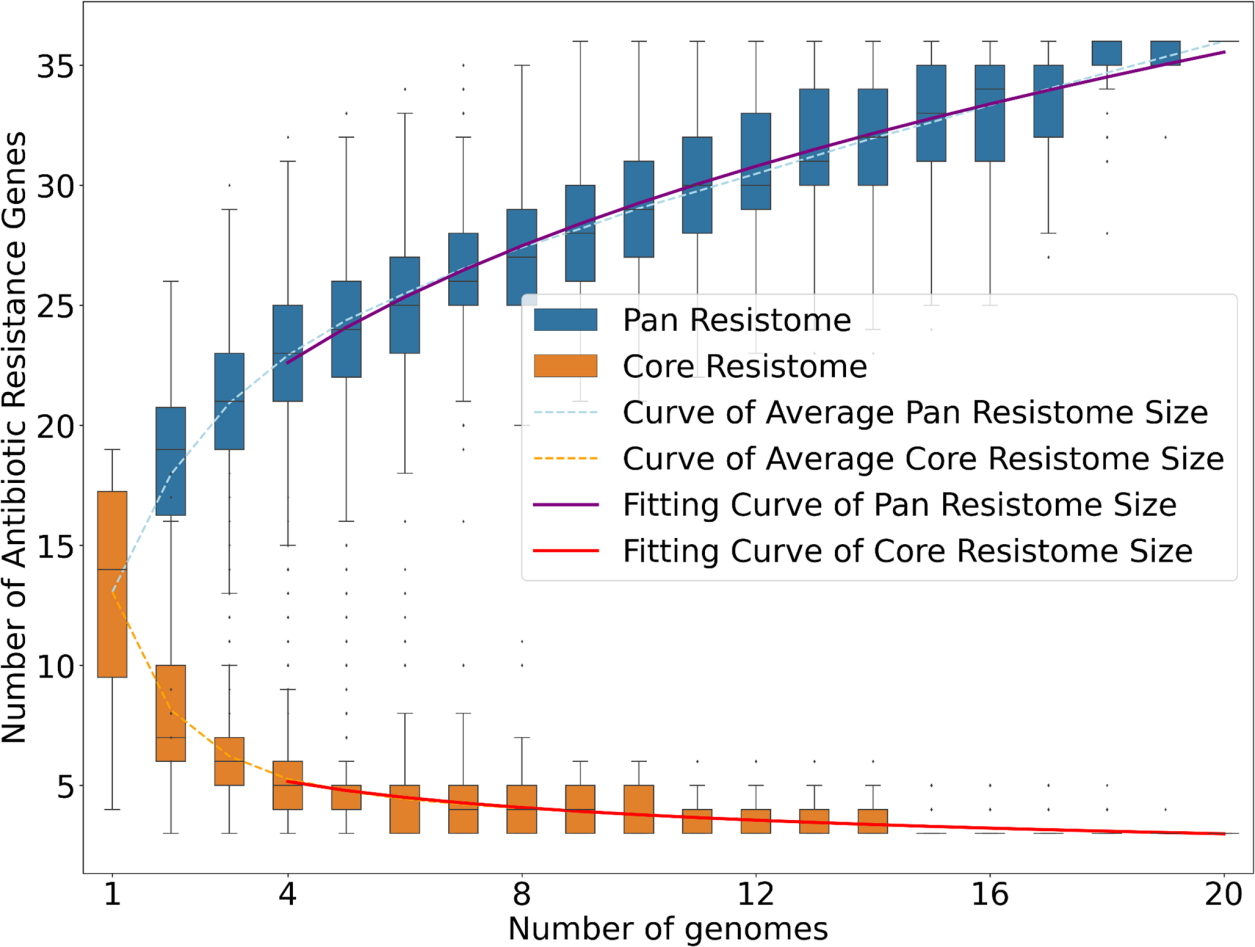
Development trends of pan-resistome and core-resistome of *Enterococcus faecium *genomes. Graph representing the pan- and core-resistome calculation by the power law. The graph presents the number of genomes on the X-axis and the number of antibiotic resistance genes on the Y-axis. The blue box plots represent the pan-resistome curve; the orange box plots represent the core-resistome curve; the blue dotted line represents the mean of the pan-resistome curve; the dotted line represents the mean of the core-resistome curve; the purple straight line represents the fitted pan-resistome curve; and the red straight line represents the fitted core-resistome curve

The PRAP analysis used the power law to calculate the pan-resistome and the core resistome. For the pan- and core-resistome, the final formulas (P = (15.317) * x^(0.2810)^ (R^2^ = 0.996) e C = (8.278) * x^(−0.340^) (R^2^ = 0.996), respectively) presented R2 values lower than 1, which suggests that this set of studied species have an open pan- and core-resistome, almost close to closing, revealing that they still can add foreign ARGs through the addition of newly sequenced genomes (Fig 7).

**Fig. 8:**
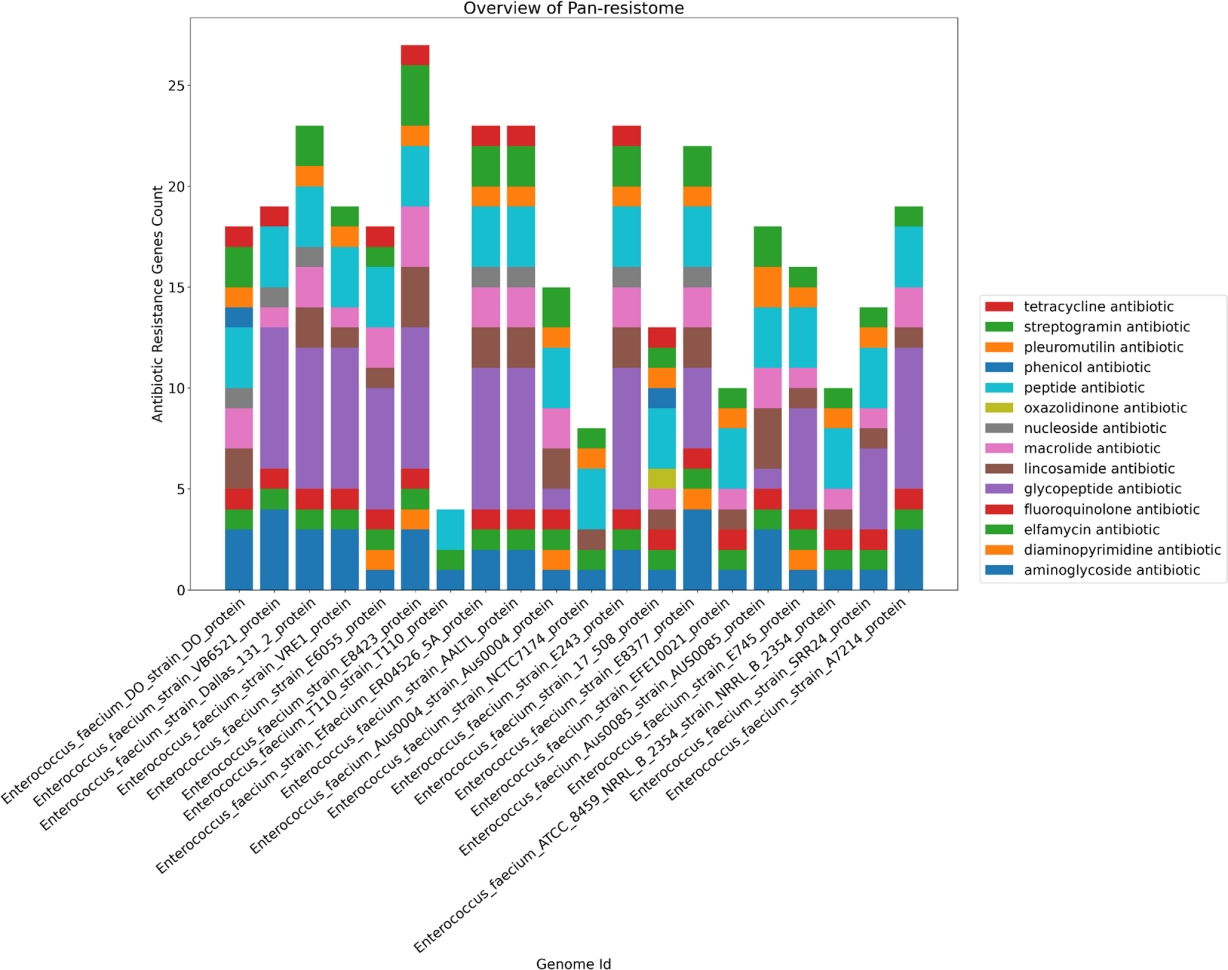
Overview of pan-resistome antibiotics. Each color in the bar graph represents the number of genes found to be resistant to the antibiotics in the legend. The genomes are represented on the X-axis and the resistance gene count for each genome is represented on the Y-axis

The following image refers to the general analysis of the pan-resistome genes, showing a range of antibiotics to which these strains are resistant (Fig. 8). There is a range of antibiotics that can be used to prevent and/or treat infections caused by this pathogen, however, the emergence and proliferation of antibiotic-resistant bacterial strains have rendered a significant number of antibiotics ineffective or only marginally effective. For this reason, constant study, as well as putting this project data on the bench, can help in a more efficient search for the resistance genes demonstrated in the project. Vertical transmission of ARGs occurs from one generation to the next, while horizontal transmission can occur between different bacterial species or strains through mobile genetic elements, such as plasmids, insertion sequences, and integrative conjugative elements [41].

All representative strains presented resistance genes to antibiotics of the Aminoglycoside, Elfamycin, and Peptide classes. Enterococci present intrinsic resistance to moderate concentrations of aminoglycosides, a characteristic observed in all species due to the limited penetration of these antimicrobials through the bacterial wall [40]. Acquired resistance to high concentrations of aminoglycoside antibiotics can result from mutations that decrease the affinity of the agent for the ribosome, as occurs with streptomycin (known as ribosomal resistance), or, more frequently, by the acquisition of new genes that encode enzymes capable of modifying aminoglycosides, a phenomenon called acquired resistance [42].

Elfamycins are a class of antibiotics that target the protein synthesis elongation factor, EF-Tu. Very important for the catalysis of bonds, they have important functions in the bacterial translation process [43]. About 20 years ago, it was an important antibiotic for differentiating between *E. faecium* and *E. faecalis*, since the former was normally susceptible and the latter, with the presence of considerable resistance [43]. In recent years, new approaches have been used to search for bacteria that carry self-resistance genes within biosynthetic clusters [44], which may be one of the explanations for why our genomes (recently sequenced) present such resistance, in line with what is described in the literature.

Peptides are typically found in a myriad of microorganisms and often exhibit broad-spectrum antimicrobial activity against many. They have become a promising alternative, given the growing resistance to traditional antibiotics [45]. In the case of *Enterococci*, we see that these microorganisms have undergone modifications to the cell wall and membrane, for example, the LiaFSR-LiaX system, which is important for mediating cell envelope remodeling, thus providing cross-resistance to these peptide antibiotics and certain bacteriocins [46].

Within the Glycopeptide class, an antibiotic of interest in the *Enterococci* literature is Vancomycin, in which 14 of 20 strains studied presented resistance genes against it. Vancomycin-resistant *Enterococci* (VRE) are mainly composed of *E. faecalis* and *E. faecium*, with the latter being frequently acquired in hospital settings. The treatment of these serious VRE infections is complex, often requiring the use of combination therapies. This is due to the fact that many effective antimicrobial agents, when used alone, demonstrate only a bacteriostatic effect [47].

In addition to the information mentioned above, one notable aspect of the T110 strain is that studies such as those by Natarajan, P., and Parani, M [48]. and Im, E., et al. [49] indicate that this strain has been and continues to be tested to determine whether it qualifies as a probiotic strain. Thus, such variations related to gene percentage, similarity to other microorganisms, and the presence of resistance or virulence genes may be acceptable and plausible.

### 5 possible drug target candidates were found

In the search for orthologous proteins, 3574 protein groups were found with the 21 genomes used in the analysis. The proteins of these groups were separated into three types of subgroups: core, shared and singletons. 1612, 1961 and 656 proteins were classified in the core, shared and singletons subgroups, respectively. The core subgroup represents the set of proteins present in all lineages, so it was selected for subsequent analyses, once the drug target has to be effective against all strains.

The BLAST tool was used to compare the core proteins found and the human proteome available from NCBI (taxid: 9606). 279 proteins had no homology against the host and were submitted for subcellular localization research, of which 30 were classified as PSE, 89 as membrane, 3 secreted, and 157 as cytoplasmic. All proteins considered cytoplasmic were analyzed for potential drug target candidates by the MHOLline program, once they are normally involved in basic processes and conserved metabolic pathways. A total of 5 proteins were filtered in the G2 structural quality subtype and are plotted in Table 2.

**Table 2:** Candidate proteins for drug targets in the human host against *Enterococcus faecium*

Protein ID	Name	Gene	MHOLline (G2 Filters Quality)	Length	Subcellular Location
WP_002287602.1	Phosphocarrier protein HPr	HPr	Very high	88 aa	Citoplasmatic
WP_002288353.1	GNAT family N-acetyltransferase	Eis	Very high	406 aa	Citoplasmatic
WP_002288695.1	Translation initiation factor IF-1	infA	Very high	72 aa	Citoplasmatic
WP_002290178.1	HU family DNA-binding protein	Hu	Very high	91 aa	Citoplasmatic
WP_002295134.1	Sugar-binding domain-containing protein	SorC	Very high	345 aa	Citoplasmatic

ID: Identity; aa: Aminoacids

The first protein of interest is HPr (WP_002287602.1), or histidine-containing phosphocarrier, a small cytoplasmic protein that plays a crucial role in the sugar transport system known as the phosphoenolpyruvate-dependent phosphotransferase (PTS) system [50]. The second protein (WP_002288353.1) is the GNAT family N-acetyltransferase, related to the N-acetyltransferase (GNAT) superfamily and associated with Gcn5. These enzymes are involved in diverse cellular activities, from antibiotic resistance to histone modifications [51]. The third (WP_002288695.1) is related to the translation initiation factor IF1, a highly conserved element of the prokaryotic translation apparatus. This factor stimulates, in vitro, the initiation phase of protein synthesis [52]. The fourth drug target is the HU family DNA-binding protein (WP_002290178.1), which is associated with DNA replication, recombination, and repair processes [53, 54]. Finally, the fifth protein (WP_002295134.1), a Sugar-binding domain-containing protein, encoded by the SorC gene, involves binding to carbohydrates, which encompasses monosaccharides, oligosaccharides, and polysaccharides, as well as derivatives resulting from the reduction of the carbonyl group (alditols) of monosaccharides, oxidation of hydroxyl groups to generate aldehydes, ketones or carboxylic acids, or the exchange of hydroxy groups for hydrogen atoms [55].

In general, cytoplasmic proteins are correlated with the essential survival processes of these organisms and can normally be found inside their cells [56]. Among these processes, all proteins found in this work play roles in enzymatic functions, structural biosynthesis, signaling, and even DNA replication and repair processes. Because these processes are vital to the microorganism, any change that occurs in the functionality of these proteins or even in their structure can be a key point for the start of a successful treatment of diseases caused by it. Therefore, as previously demonstrated in the literature, these targets are strongly considered potential candidates for drug targets, which favors drug research more quickly than conventional methods [11, 57].

### Drug targets and ligands interactions

The information regarding the top 10 interactions between drug targets and the ZINC compounds is presented in Table S2. The strongest interactions are highlighted in yellow.

**Fig. 9:**
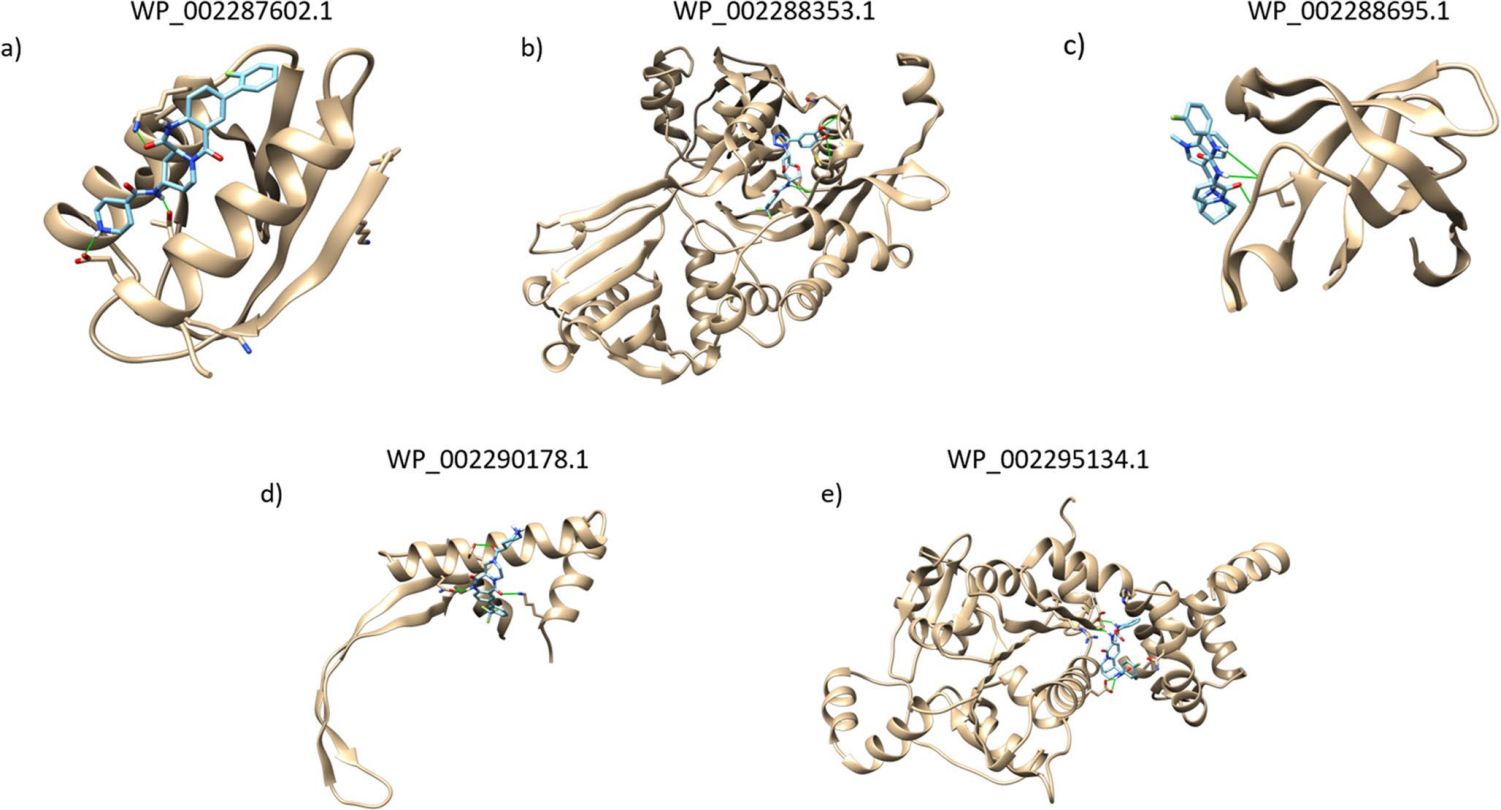
Docking interactions among proteins and ZINC Compound. Each 3D image represents the proteins interacting with your best ZINC Compound. The proteins are colored in gold, and the compound in shades of blue. The link interaction is a green line among the molecules

For the first drug target, WP_002287602.1, the best interaction was observed with ZINC03840479, showing three bonding interactions (LYS28, THR80, and GLU84) and a binding affinity of −7.074. The next target, WP_002288353.1, showed the strongest interaction with ZINC04270543, presenting four bonding interactions (VAL97, THR99, ASN105, and MET109) and a binding affinity of −10.33. The protein WP_002288695.1 interacted with ZINC03842061, with three bonding interactions (ILE47) and a binding affinity of −7.78. WP_002290178.1 showed interaction with ZINC04235972, forming three bonds (LYS4, SER31, and GLN34) and a binding affinity of −7.869. Lastly, the protein WP_002295134.1 interacted with ZINC04259094, forming four bonds (GLU98, VAL112, and GLU133 twice) and a binding affinity of −9.12 (Fig. 9).

In general, ZINC compounds are mainly composed of natural products, which are chemical substances produced by living organisms. These include small molecules involved in metabolic reactions, whether essential or non-essential for survival. Due to these characteristics, natural products are frequently used in virtual screening studies, either for drug discovery or for identifying biological activity. Additionally, they are generally more accessible and cost-effective in modern pharmaceutical research [58].

### Limitations of work

This study initially involves an analysis of a small cluster of selected genomes from clades on the NCBI phylogenetic tree. Therefore, it cannot be definitively stated that all the genes identified here are present in other lineages of the same species; consequently, this information requires confirmation. Furthermore, since this is an in silico study, all findings presented here are theoretical in nature, intended to advance our understanding of the microorganism in question and help reduce the time required to develop new therapeutic treatments; consequently, the information presented here requires in vitro and in vivo confirmation. The research group is addressing all of these limitations, and they are expected to be resolved soon in new publications on the subject.

## Conclusions

The study on the *Enterococcus* genus, with a focus on *E. faecium*, highlighted its clinical relevance and complexity, particularly in the context of nosocomial infections and antimicrobial resistance. Pan-genomic and phylogenetic analyses revealed remarkable genetic diversity, characterized by genomic rearrangements, horizontal gene transfer, and the presence of pathogenicity and resistance islands, which contribute to the adaptation and persistence of this pathogen in hospital environments. The identification of antibiotic resistance genes, including those associated with vancomycin resistance, underscores the urgent need for novel antimicrobial therapies and combination treatment strategies.

The application of reverse vaccinology and orthologous protein analysis identified five essential cytoplasmic proteins as potential therapeutic targets. These proteins, involved in critical processes such as sugar transport, histone modification, translation initiation, and DNA repair, represent promising targets for the development of innovative therapeutic approaches against *E. faecium*.

These findings emphasize the importance of continuous genomic surveillance and advancements in the identification of novel therapeutic targets to combat antimicrobial resistance. Strategies such as prudent antibiotic use, strengthened infection control measures, and investment in research focused on comparative genomics and molecular biology are crucial to addressing the challenges posed by *E. faecium*. Integrating insights into resistance and virulence mechanisms with the development of targeted therapies could significantly reduce the morbidity and mortality associated with infections caused by this pathogen.

## Electronic Supplementary Material

Below is the link to the electronic supplementary material.

Supplementary Material 1 (DOCX 335 KB)

## Data Availability

The genomes are available at Refseq as described in Table 1.

## References

[1] Wang Y, Xu W, Guo S et al (2025) Enterococci for human health: a friend or foe? Microb Pathog 201:107381 39983880 10.1016/j.micpath.2025.107381

[2] Murray BE (1990) The life and times of the Enterococcus. Clin Microbiol Rev 3(1):46–65 2404568 10.1128/cmr.3.1.46 PMC358140

[3] Gao W, Howden BP, Stinear TP (2018) Evolution of virulence in Enterococcus faecium, a hospital-adapted opportunistic pathogen. Curr Opin Microbiol 41:76–82 29227922 10.1016/j.mib.2017.11.030

[4] Furlaneto-Maia L, Rocha KR, Siqueira VLD, Furlaneto MC (2014) Comparison between automated system and PCR-based method for identification and antimicrobial susceptibility profile of clinical *Enterococcus* spp. Rev Inst Med Trop Sao Paulo 56(2):97–103 24626409 10.1590/S0036-46652014000200002 PMC4085851

[5] Rice LB (2008) Federal Funding for the Study of Antimicrobial Resistance in Nosocomial Pathogens: No ESKAPE. J Infect Dis 197(8):1079–1081 18419525 10.1086/533452

[6] Gorrie C, Higgs C, Carter G, Stinear TP, Howden B (2019) Genomics of vancomycin-resistant Enterococcus faecium. Microb Genom. ;5(7) 10.1099/mgen.0.000283 PMC6700659 31329096

[7] Miller WR, Murray BE, Rice LB, Arias CA (2020) Resistance in Vancomycin-Resistant Enterococci. Infect Dis Clin North Am 34(4):751–771 33131572 10.1016/j.idc.2020.08.004 PMC7640809

[8] Zhou X, Willems RJL, Friedrich AW, Rossen JWA, Bathoorn E (2020) Enterococcus faecium: from microbiological insights to practical recommendations for infection control and diagnostics. Antimicrob Resist Infect Control 9(1):130 32778149 10.1186/s13756-020-00770-1 PMC7418317

[9] Medini D, Donati C, Tettelin H, Masignani V, Rappuoli R (2005) The microbial pan-genome. Curr Opin Genet Dev 15(6):589–594 16185861 10.1016/j.gde.2005.09.006

[10] Hansen EE, Lozupone CA, Rey FE, Wu M, Guruge JL, Narra A et al (2011) Pan-genome of the dominant human gut-associated archaeon, *Methanobrevibacter smithii*, studied in twins. Proc Natl Acad Sci U S A 108(SUPPL. 1):4599–606 21317366 10.1073/pnas.1000071108 PMC3063581

[11] Barh D, Tiwari S, Jain N, Ali A, Santos AR, Misra AN et al (2011) In silico subtractive genomics for target identification in human bacterial pathogens. Drug Dev Res 72(2):162–177

[12] Ågren J, Sundström A, Håfström T, Segerman B (2012) Gegenees: fragmented alignment of multiple genomes for determining phylogenomic distances and genetic signatures unique for specified target groups. PLoS One. 10.1371/journal.pone.0039107 22723939 10.1371/journal.pone.0039107 PMC3377601

[13] Huson DH, Bryant D (2006) Application of phylogenetic networks in evolutionary studies. Mol Biol Evol 23(2):254–67. 10.1093/molbev/msj030 16221896 10.1093/molbev/msj030

[14] Kim M, Oh HS, Park SC, Chun J (2014) Towards a taxonomic coherence between average nucleotide identity and 16S rRNA gene sequence similarity for species demarcation of prokaryotes. Int J Syst Evol Microbiol 64(Pt2):346–351 24505072 10.1099/ijs.0.059774-0

[15] Darling AE, Mau B, Perna NT, Progressivemauve (2010) Multiple genome alignment with gene gain, loss and rearrangement. PLoS One [Internet]. ;5(6). Available from: http://dx.plos.org/10.1371/journal.pone.0011147 10.1371/journal.pone.0011147 PMC2892488 20593022

[16] Soares SC, Geyik H, Ramos RTJ, de Sá PHCG, Barbosa EGV, Baumbach J et al (2016) GIPSy: genomic island prediction software. J Biotechnol 232:2–11 26376473 10.1016/j.jbiotec.2015.09.008

[17] Alikhan NF, Petty NK, Ben Zakour NL, Beatson SA (2011) BLAST Ring Image Generator (BRIG): simple prokaryote genome comparisons. BMC Genomics. 10.1186/1471-2164-12-402 21824423 10.1186/1471-2164-12-402 PMC3163573

[18] He Y, Zhou X, Chen Z, Deng X, Gehring A, Ou H et al (2020) PRAP: Pan Resistome analysis pipeline. BMC Bioinformatics 21(1):20 31941435 10.1186/s12859-019-3335-y PMC6964052

[19] Emms DM, Kelly S (2015) OrthoFinder: solving fundamental biases in whole genome comparisons dramatically improves orthogroup inference accuracy. Genome Biol. 10.1186/s13059-015-0721-2 26243257 10.1186/s13059-015-0721-2 PMC4531804

[20] Camacho C, Coulouris G, Avagyan V, Ma N, Papadopoulos J, Bealer K et al (2009) BLAST+: architecture and applications. BMC Bioinformatics 10(1):421 20003500 10.1186/1471-2105-10-421 PMC2803857

[21] Barinov A, Loux V, Hammani A, Nicolas P, Langella P, Ehrlichh D et al (2009) Prediction of surface exposed proteins in *Streptococcus pyogenes*, with a potential application to other Gram-positive bacteria. Proteomics 9(1):61–73. 10.1002/pmic.200800195 19053137 10.1002/pmic.200800195

[22] Capriles PVSZ, Guimarães ACR, Otto TD, Miranda AB, Dardenne LE, Degrave WM (2010) Structural modelling and comparative analysis of homologous, analogous and specific proteins from *Trypanosoma cruzi* versus *Homo sapiens*: putative drug targets for Chagas’ disease treatment. BMC Genomics. 10.1186/1471-2164-11-610 21034488 10.1186/1471-2164-11-610 PMC3091751

[23] UniProt (2019) UniProt: a worldwide hub of protein knowledge. Nucleic Acids Res [Internet]. ;47(D1):D506–15. Available from: https://academic.oup.com/nar/article/47/D1/D506/5160987 10.1093/nar/gky1049 PMC6323992 30395287

[24] Cavasotto CN, Aucar MG (2020) High-throughput docking using quantum mechanical scoring. Front Chem. 10.3389/fchem.2020.00246 32373579 10.3389/fchem.2020.00246 PMC7186494

[25] Kumar M, Rathore RS (2025) *RamPlot*: a webserver to draw 2D, 3D and assorted Ramachandran (φ, ψ) maps. J Appl Crystallogr 58(2):630–636

[26] Garrett MM, Ruth H, William L, Michel FS, Richard KB, G DS et al (2009) AutoDock4 and AutoDockTools4: automated docking with selective receptor flexibility. J Comput Chem 30(16):2785–5791. 10.1002/jcc.21256 19399780 10.1002/jcc.21256 PMC2760638

[27] Fährrolfes R, Bietz S, Flachsenberg F, Meyder A, Nittinger E, Otto T et al (2017) Proteins Plus: A web portal for structure analysis of macromolecules. Nucleic Acids Res [Internet]. ;45(W1):W337–43. Available from: https://www.ncbi.nlm.nih.gov/pubmed/28472372 10.1093/nar/gkx333 PMC5570178 28472372

[28] Rodrigues TCV, Jaiswal AK, De Sarom A, Oliveira LDC, Oliveira CJF, Ghosh P et al (2019) Reverse vaccinology and subtractive genomics reveal new therapeutic targets against *Mycoplasma pneumoniae*: a causative agent of pneumonia. R Soc Open Sci. 10.1098/rsos.190907 10.1098/rsos.190907 PMC6689572 31417766

[29] Oleg T, Arthur JO (2010) AutoDock Vina: improving the speed and accuracy of docking with a new scoring function, efficient optimization, and multithreading. J Comput Chem 31(2):455–61. 10.1002/jcc.21334 19499576 10.1002/jcc.21334 PMC3041641

[30] Pettersen EF, Goddard TD, Huang CC, Couch GS, Greenblatt DM, Meng EC et al (2004) UCSF Chimera - a visualization system for exploratory research and analysis. J Comput Chem 25(13):1605–12. 10.1002/jcc.20084 15264254 10.1002/jcc.20084

[31] Top J, Arredondo-Alonso S, Schürch AC, Puranen S, Pesonen M, Pensar J et al (2020) Genomic rearrangements uncovered by genome-wide co-evolution analysis of a major nosocomial pathogen, Enterococcus faecium. Microb Genom. ;6(12) 10.1099/mgen.0.000488 PMC8116687 33253085

[32] Silva-Pereira TT, Soler-Camargo NC, Guimarães AMS (2024) Diversification of gene content in the *Mycobacterium tuberculosis* complex is determined by phylogenetic and ecological signatures. Microbiol Spectr. ;12(2) 10.1128/spectrum.02289-23 PMC10871547 38230932

[33] Dong Y, Chen S, Cheng S, Zhou W, Ma Q, Chen Z et al (2019) Natural selection and repeated patterns of molecular evolution following allopatric divergence. Elife. ;8 10.7554/eLife.45199 PMC6744222 31373555

[34] Tokuda M, Shintani M (2024) Microbial evolution through horizontal gene transfer by mobile genetic elements. Microb Biotechnol. ;17(1) 10.1111/1751-7915.14408 PMC10832538 38226780

[35] van Hal SJ, Ip CLC, Ansari MA, Wilson DJ, Espedido BA, Jensen SO et al (2016) Evolutionary dynamics of Enterococcus faecium reveals complex genomic relationships between isolates with independent emergence of vancomycin resistance. Microb Genom. ;2(1) 10.1099/mgen.0.000048 PMC5049587 27713836

[36] Simon C (2022) An Evolving View of Phylogenetic Support. Syst Biol 71(4):921–928 32915964 10.1093/sysbio/syaa068

[37] Schwartzman JA, Lebreton F, Salamzade R, Shea T, Martin MJ, Schaufler K et al (2024) Global diversity of enterococci and description of 18 previously unknown species. Proceedings of the National Academy of Sciences. ;121(10) 10.1073/pnas.2310852121 PMC10927581 38416678

[38] Getachew Y, Hassan L, Zakaria Z, Abdul Aziz S (2013) Genetic Variability of Vancomycin-Resistant Enterococcus faecium and Enterococcus faecalis Isolates from Humans, Chickens, and Pigs in Malaysia. Appl Environ Microbiol 79(15):4528–4533 23666337 10.1128/AEM.00650-13 PMC3719509

[39] García-Solache M, Rice LB (2019) The Enterococcus: a Model of Adaptability to Its Environment. Clin Microbiol Rev. ;32(2) 10.1128/CMR.00058-18 PMC6431128 30700430

[40] Kristich CJ, Rice LB, Arias CA (2014) Enterococcal Infection—Treatment and Antibiotic Resistance

[41] Li J, Tai C, Deng Z, Zhong W, He Y, Ou HY (2017) VRprofile: gene-cluster-detection-based profiling of virulence and antibiotic resistance traits encoded within genome sequences of pathogenic bacteria. Brief Bioinform. ;bbw141 10.1093/bib/bbw141 28077405

[42] Simner PJ, Humphries RM (2023) Special Phenotypic Methods for Detecting Antibacterial Resistance. ClinMicroNow. Wiley, pp 1–34

[43] Miele A, Goldstein BP, Bandera M, Jarvis C, Resconi A, Williams RJ (1994) Differential susceptibilities of enterococcal species to elfamycin antibiotics. J Clin Microbiol 32(8):2016–2018 7989561 10.1128/jcm.32.8.2016-2018.1994 PMC263923

[44] Yarlagadda V, Medina R, Johnson TA, Koteva KP, Cox G, Thaker MN et al (2020) Resistance-Guided Discovery of Elfamycin Antibiotic Producers with Antigonococcal Activity. ACS Infect Dis 6(12):3163–3173 33164482 10.1021/acsinfecdis.0c00467

[45] Rima M, Rima M, Fajloun Z, Sabatier JM, Bechinger B, Naas T (2021) Antimicrobial Peptides: A Potent Alternative to Antibiotics. Antibiotics 10(9):1095 34572678 10.3390/antibiotics10091095 PMC8466391

[46] Tymoszewska A, Szylińska M, Aleksandrzak-Piekarczyk T (2023) The LiaFSR-LiaX System Mediates Resistance of Enterococcus faecium to Peptide Antibiotics and to Aureocin A53- and Enterocin L50-Like Bacteriocins. Microbiol Spectr. ;11(3) 10.1128/spectrum.00343-23 PMC10269926 37219451

[47] Rubinstein E, Keynan Y (2013) Vancomycin-Resistant Enterococci. Crit Care Clin 29(4):841–852 24094380 10.1016/j.ccc.2013.06.006

[48] Natarajan P, Parani M (2015) First complete genome sequence of a probiotic *Enterococcus faecium* strain T-110 and its comparative genome analysis with pathogenic and non-pathogenic *Enterococcus faecium* genomes. J Genet Genomics 42:43–6. 10.1016/j.jgg.2014.07.002 25619602 10.1016/j.jgg.2014.07.002

[49] Im EJ, Lee H-Y, Kim M, Kim M-K (2023) Evaluation of enterococcal probiotic usage and review of potential health benefits, safety, and risk of antibiotic-resistant strain emergence. Antibiotics Basel 12:1327. 10.3390/antibiotics12081327 37627747 10.3390/antibiotics12081327 PMC10451534

[50] Bender JK, Fiedler S, Klare I, Werner G (2015) Complete Genome Sequence of the Gut Commensal and Laboratory Strain Enterococcus faecium 64/3. Genome Announc. ;3(6) 10.1128/genomeA.01275-15 PMC4653773 26586871

[51] Wagner TM, Janice J, Sivertsen A, Sjögren I, Sundsfjord A, Hegstad K (2021) Alternative vanHAX promoters and increased vanA -plasmid copy number resurrect silenced glycopeptide resistance in Enterococcus faecium. J Antimicrob Chemother 76(4):876–882 33367710 10.1093/jac/dkaa541 PMC7953315

[52] Cummings HS, Hershey JW (1994) Translation initiation factor IF1 is essential for cell viability in Escherichia coli. J Bacteriol 176(1):198–205 8282696 10.1128/jb.176.1.198-205.1994 PMC205031

[53] Swinger KK, Rice PA (2007) Structure-based Analysis of HU–DNA Binding. J Mol Biol 365(4):1005–1016 17097674 10.1016/j.jmb.2006.10.024 PMC1945228

[54] Grove A (2011) Functional Evolution of Bacterial Histone-Like HU Proteins. Curr Issues Mol Biol 13:1–12 20484776

[55] de Sanctis D, McVey CE, Enguita FJ, Carrondo MA (2009) Crystal structure of the full-length sorbitol operon regulator SorC from *Klebsiella pneumoniae*: structural evidence for a novel transcriptional regulation mechanism. J Mol Biol 387(3):759–70 19232357 10.1016/j.jmb.2009.02.017

[56] Van Tartwijk FW, Kaminski CF (2022) Protein Condensation, Cellular Organization, and Spatiotemporal Regulation of Cytoplasmic Properties. Adv Biol. ;6(11) 10.1002/adbi.202101328 35796197

[57] Barh D, Kumar A (2009) In silico Identification of Candidate Drug and Vaccine Targets from Various Pathways in Neisseria gonorrhoeae. Silico Biology: J Biol Syst Model Multi-Scale Simul 9(4):225–231 20109152

[58] Sorokina M, Steinbeck C (2020) Review on natural products databases: where to find data in 2020. J Cheminform 12(1):20 33431011 10.1186/s13321-020-00424-9 PMC7118820

