# Supplementary material for: Pangenomics insights of enterococcus faecium human isolates and identification of novel therapeutic targets by in silico subtractive genomics
Source: Braz J Microbiol. 2026 Jun 3;57(1):166. doi: 10.1007/s42770-026-01983-z (PMC13234099; doi:10.1007/s42770-026-01983-z)
Supplement: Supplementary file 1 — Supplementary Material 1 (DOCX 335 KB) [file 42770_2026_1983_MOESM1_ESM.docx]

**Table S1: Genomics islands localizations**

| *E. casseliflavus EC20* | | |  | *E. casseliflavus EC20* | | |
| --- | --- | --- | --- | --- | --- | --- |
| GEI | Start | Stop |  | GEI | Start | Stop |
| PAI 1 | 216064 | 225401 |  | GEI 1 | 225401 | 216064 |
| GEI 1 | 767559 | 810768 |  | GEI 2 | 307233 | 253653 |
| PAI 2 | 814300 | 849764 |  | GEI 3 | 639861 | 630340 |
| GEI 2 | 937823 | 1005424 |  | GEI 4 | 668712 | 647398 |
| GEI 3 | 1087931 | 1111770 |  | GEI 5 | 810768 | 767559 |
| PAI 3 | 1717927 | 1739396 |  | RI 1 | 849764 | 814300 |
| PAI 4 | 1743491 | 1757735 |  | GEI 6 | 1005424 | 937823 |
| GEI 4 | 1782639 | 1789814 |  | RI 2 | 1111770 | 1087931 |
| GEI 5 | 1990831 | 2031726 |  | GEI 7 | 1739396 | 1717927 |
| PAI 5 | 2050193 | 2065773 |  | GEI 8 | 1789814 | 1782639 |
| PAI 6 | 2108910 | 2119577 |  | GEI 9 | 2031726 | 1990831 |
| PAI 7 | 2130523 | 2139154 |  | RI 3 | 2065773 | 2050193 |
| GEI 6 | 2222221 | 2332303 |  | RI 4 | 2119577 | 2108910 |
| PAI 8 | 2659091 | 2721113 |  | RI 5 | 2139154 | 2130523 |
|  |  |  |  | GEI 10 | 2332303 | 2222221 |
|  |  |  |  | GEI 11 | 2359724 | 2354523 |
|  |  |  |  | RI 6 | 2721113 | 2659091 |

| *E. gallinarum FDAARGOS-163* | | |  | *E. gallinarum FDAARGOS-163* | | |
| --- | --- | --- | --- | --- | --- | --- |
| GEI | Start | Stop |  | GEI | Start | Stop |
| PAI 1 | 225401 | 205806 |  | GEI 1 | 138388 | 127714 |
| PAI 2 | 268297 | 253653 |  | GEI 2 | 225401 | 205806 |
| GEI 1 | 668712 | 646328 |  | GEI 3 | 26829 | 253653 |
| GEI 2 | 808535 | 767559 |  | GEI 4 | 639861 | 630340 |
| PAI 3 | 849764 | 819234 |  | GEI 5 | 668712 | 646328 |
| PAI 4 | 1005424 | 953445 |  | GEI 6 | 808535 | 767559 |
| GEI 3 | 1111770 | 1087931 |  | RI 1 | 84976 | 819234 |
| GEI 4 | 1214613 | 1201791 |  | RI 2 | 1005424 | 953445 |
| PAI 5 | 1739396 | 1717927 |  | RI 3 | 1111770 | 1087931 |
| PAI 6 | 1757735 | 1743491 |  | GEI 7 | 1214613 | 1201791 |
| PAI 7 | 1792193 | 1782639 |  | GEI 8 | 173939 | 1717927 |
| GEI 5 | 2031726 | 1991285 |  | RI 4 | 1792193 | 1782639 |
| PAI 8 | 2065773 | 2050193 |  | GEI 9 | 2031726 | 1991285 |
| PAI 9 | 2139154 | 2108910 |  | RI 5 | 2065773 | 2050193 |
| PAI 10 | 2206620 | 2194626 |  | RI 6 | 2139154 | 2108910 |
| GEI 6 | 2299499 | 2224762 |  | GEI 10 | 2299499 | 2224762 |
| PAI 11 | 2332303 | 2303040 |  | GEI 11 | 2332303 | 2303040 |
| PAI 12 | 2741120 | 2659091 |  | GEI 12 | 2359724 | 2354523 |
| GEI 7 | 2756281 | 2748469 |  | GEI 13 | 2741120 | 2659091 |

Note: PAI = Pathogenicity Island; RI = Resistance Island; GEI = Genomic Island.

**Table S2: Docking among Drug targets and ZINC Natural Compounds**

| **Protein Target ID** | **Protein Name** | **ZINC Compound ID** | **Pose/Autodock Vina Binding Affinity (Kcal/mol)** | **Number of Hydrogen** | **Bond Interaction** |
| --- | --- | --- | --- | --- | --- |
| WP_002287602.1 | Phosphocarrier protein HPr | DLNC_ZINC03840479 | 1/-7.728 | 0 | No Interaction |
|  |  |  | 2/-7.074 | 3 | LYS 28, THR 80, GLU 84 |
|  |  |  | 3/-6.894 | 0 | No Interaction |
|  |  |  | 4/-6.737 | 2 | LYS 3, MET 1 |
|  |  |  | 5/-6.558 | 1 | LYS 82 |
|  |  | DLNC_ZINC04222214 | 1/-7.52 | 0 | No Interaction |
|  |  |  | 2/-7.193 | 0 | No Interaction |
|  |  |  | 3/-7.146 | 1 | LYS 28 |
|  |  |  | 4/-7.042 | 0 | No Interaction |
|  |  |  | 5/-7.023 | 0 | No Interaction |
|  |  | DLNC_ZINC04235928 | 1/-7.917 | 1 | ASN 30 |
|  |  |  | 2/-7.195 | 0 | No Interaction |
|  |  |  | 3/-6.879 | 1 | THR 12 |
|  |  |  | 4/-6.735 | 1 | HIS 7 |
|  |  |  | 5/-6.695 | 0 | No Interaction |
|  |  | DLNC_ZINC04235972 | 1/-7.536 | 0 | No Interaction |
|  |  |  | 2/-7.038 | 1 | THR 80 |
|  |  |  | 3/-6.834 | 1 | THR 12 |
|  |  |  | 4/-6.787 | 1 | THR 12 |
|  |  |  | 5/-6.764 | 0 | No Interaction |
|  |  | DLNC_ZINC04236028 | 1/-7.579 | 1 | LYS 83 |
|  |  |  | 2/-7.118 | 0 | No Interaction |
|  |  |  | 3/-6.804 | 0 | No Interaction |
|  |  |  | 4/-6.717 | 1 | THR 80 |
|  |  |  | 5/-6.664 | 0 | No Interaction |
|  |  | DLNC_ZINC04236030 | 1/-7.667 | 1 | LYS 83 |
|  |  |  | 2/-7.326 | 1 | LYS 28 |
|  |  |  | 3/-7.259 | 0 | No Interaction |
|  |  |  | 4/-6.938 | 1 | GLN 24 |
|  |  |  | 5/-6.709 | 0 | No Interaction |
|  |  | DLNC_ZINC04236083 | 1/-7.588 | 0 | No Interaction |
|  |  |  | 2/-6.57 | 0 | No Interaction |
|  |  |  | 3/-6.568 | 0 | No Interaction |
|  |  |  | 4/-6.322 | 0 | No Interaction |
|  |  |  | 5/-6.251 | 0 | No Interaction |
|  |  | DLNC_ZINC04236421 | 1/-7.791 | 0 | No Interaction |
|  |  |  | 2/-7.183 | 0 | No Interaction |
|  |  |  | 3/-7.082 | 0 | No Interaction |
|  |  |  | 4/-7.05 | 0 | No Interaction |
|  |  |  | 5/-6.78 | 1 | THR 12 |
|  |  | DLNC_ZINC04260398 | 1/-7.547 | 0 | No Interaction |
|  |  |  | 2/-7.048 | 0 | No Interaction |
|  |  |  | 3/-6.711 | 0 | No Interaction |
|  |  |  | 4/-6.634 | 0 | No Interaction |
|  |  |  | 5/-6.579 | 0 | No Interaction |
|  |  | DLNC_ZINC67902338 | 1/-7.557 | 0 | No Interaction |
|  |  |  | 2/-6.633 | 1 | LYS 45 |
|  |  |  | 3/-6.565 | 1 | THR 80 |
|  |  |  | 4/-6.541 | 0 | No Interaction |
|  |  |  | 5/-6.509 | 2 | THR 12, GLU 11 |
| WP_002288353.1 | Enhanced intracellular survival protein Eis | DLNC_ZINC05415069 | 1/-11.54 | 0 | No interaction |
|  |  |  | 2/-8.92 | 1 | LYS 149 |
|  |  |  | 3/-8.596 | 0 | No interaction |
|  |  |  | 4/-8.281 | 0 | No interaction |
|  |  |  | 5/-8.036 | 0 | No interaction |
|  |  | DLNC_ZINC06131115 | 1/-11.44 | 1 | TYR 294 |
|  |  |  | 2/-10.62 | 1 | LYS 241 |
|  |  |  | 3/-10.27 | 1 | GLU 40 |
|  |  |  | 4/-10.12 | 0 | No interaction |
|  |  |  | 5/-9.479 | 1 | THR 99 |
|  |  | DLNC_ZINC05415064 | 1/-11.43 | 1 | LYS 149 |
|  |  |  | 2/-9.209 | 2 | TYR 273. LYS 149 |
|  |  |  | 3/-9.016 | 0 | No interaction |
|  |  |  | 4/-8.926 | 0 | No interaction |
|  |  |  | 5/-8.73 | 0 | No interaction |
|  |  | DLNC_ZINC04278011 | 1/-11.43 | 0 | No interaction |
|  |  |  | 2/-10.53 | 1 | TYR 405 |
|  |  |  | 3/-9.952 | 1 | THR 99 |
|  |  |  | 4/-9.764 | 2 | GLU 40, GLU 40 |
|  |  |  | 5/-9.749 | 1 | THR 99 |
|  |  | DLNC_ZINC05415832 | 1/-11.4 | 1 | LYS 149 |
|  |  |  | 2/-9.622 | 0 | No interaction |
|  |  |  | 3/-9.276 | 0 | No interaction |
|  |  |  | 4/-9.065 | 0 | No interaction |
|  |  |  | 5/-8.727 | 0 | No interaction |
|  |  | DLNC_ZINC04235924 | 1/-11.4 | 0 | No interaction |
|  |  |  | 2/-10.61 | 0 | No interaction |
|  |  |  | 3/-10.43 | 2 | TYR 294. LYS 241 |
|  |  |  | 4/-10.35 | 0 | No interaction |
|  |  |  | 5/-9.119 | 0 | No interaction |
|  |  | DLNC_ZINC04235880 | 1/-11.35 | 1 | VAL 97 |
|  |  |  | 2/-10.55 | 0 | No interaction |
|  |  |  | 3/-9.836 | 0 | No interaction |
|  |  |  | 4/-8.538 | 2 | GLN 32, GLY 107 |
|  |  |  | 5/-8.501 | 2 | THR 99, VAL 30 |
|  |  | DLNC_ZINC05415084 | 1/-11.34 | 1 | LYS 149 |
|  |  |  | 2/-8.945 | 1 | THR 99 |
|  |  |  | 3/-8.641 | 0 | No interaction |
|  |  |  | 4/-8.336 | 0 | No interaction |
|  |  |  | 5/-8.31 | 0 | No interaction |
|  |  | DLNC_ZINC04270543 | 1/-11.34 | 0 | No interaction |
|  |  |  | 2/-10.33 | 4 | VAL 97, ASN 105, MET 109, THR 99 |
|  |  |  | 3/-9.818 | 0 | No interaction |
|  |  |  | 4/-9.625 | 0 | No interaction |
|  |  |  | 5/-9.38 | 2 | THR 99, VAL 97 |
|  |  | DLNC_ZINC15709489 | 1/-11.3 | 0 | No interaction |
|  |  |  | 2/-10.91 | 0 | No interaction |
|  |  |  | 3/-10.07 | 0 | No interaction |
|  |  |  | 4/-9.783 | 1 | LYS 241 |
|  |  |  | 5/-9.575 | 0 | No interaction |
| WP_002288695.1 | translation initiation factor IF-1 | DLNC_ZINC03839953 | 1/-8.36 | 0 | No interaction |
|  |  |  | 2/-7.672 | 1 | THR 16 |
|  |  |  | 3/-7.359 | 1 | THR 16 |
|  |  |  | 4/-6.918 | 1 | THR 16 |
|  |  |  | 5/-6.729 | 0 | No interaction |
|  |  | DLNC_ZINC03842061 | 1/-8.317 | 0 | No interaction |
|  |  |  | 2/-7.78 | 3 | ILE 47, ILE 47, ILE 47 |
|  |  |  | 3/-7.761 | 0 | No interaction |
|  |  |  | 4/-7.736 | 1 | SER 58 |
|  |  |  | 5/-7.637 | 0 | No interaction |
|  |  | DLNC_ZINC04235972 | 1/-8.693 | 1 | THR 16 |
|  |  |  | 2/-8.512 | 1 | THR 16 |
|  |  |  | 3/-7.973 | 1 | ALA 20 |
|  |  |  | 4/-7.743 | 0 | No interaction |
|  |  |  | 5/-7.702 | 1 | THR 16 |
|  |  | DLNC_ZINC04237082 | 1/-8.308 | 0 | No interaction |
|  |  |  | 2/-7.62 | 1 | THR 68 |
|  |  |  | 3/-7.387 | 0 | No interaction |
|  |  |  | 4/-7.375 | 0 | No interaction |
|  |  |  | 5/-7.311 | 0 | No interaction |
|  |  | DLNC_ZINC04237091 | 1/-8.252 | 0 | No interaction |
|  |  |  | 2/-7.784 | 2 | THR 16, THR 16 |
|  |  |  | 3/-7.499 | 0 | No interaction |
|  |  |  | 4/-7.498 | 0 | No interaction |
|  |  |  | 5/-7.465 | 2 | ASN 19, ALA 20 |
|  |  | DLNC_ZINC04277685 | 1/-8.298 | 0 | No interaction |
|  |  |  | 2/-8.109 | 1 | THR 68 |
|  |  |  | 3/-7.861 | 1 | ALA 20 |
|  |  |  | 4/-7.354 | 1 | THR 16 |
|  |  |  | 5/-7.192 | 0 | No interaction |
|  |  | DLNC_ZINC05415069 | 1/-8.331 | 0 | No interaction |
|  |  |  | 2/-7.934 | 0 | No interaction |
|  |  |  | 3/-7.849 | 0 | No interaction |
|  |  |  | 4/-7.318 | 0 | No interaction |
|  |  |  | 5/-7.269 | 0 | No interaction |
|  |  | DLNC_ZINC08300419 | 1/-8.256 | 0 | No interaction |
|  |  |  | 2/-8.004 | 0 | No interaction |
|  |  |  | 3/-7.778 | 2 | LYS 3, LYS 39 |
|  |  |  | 4/-7.497 | 0 | No interaction |
|  |  |  | 5/-7.322 | 0 | No interaction |
|  |  | DLNC_ZINC20503625 | 1/-8.635 | 0 | No interaction |
|  |  |  | 2/-6.786 | 0 | No interaction |
|  |  |  | 3/-6.712 | 1 | ALA 20 |
|  |  |  | 4/-6.685 | 1 | ILE 47 |
|  |  |  | 5/-6.453 | 0 | No interaction |
|  |  | DLNC_ZINC20503640 | 1/-8.7 | 0 | No interaction |
|  |  |  | 2/-8.401 | 0 | No interaction |
|  |  |  | 3/-7.808 | 1 | THR 35 |
|  |  |  | 4/-7.533 | 1 | ALA 20 |
|  |  |  | 5/-7.439 | 0 | No interaction |
| WP_002290178.1 | HU family DNA-binding protein | DLNC_ZINC04235972 | 1/-8.648 | 0 | No interaction |
|  |  |  | 2/-8.433 | 0 | No interaction |
|  |  |  | 3/-8.211 | 0 | No interaction |
|  |  |  | 4/-7.913 | 0 | No interaction |
|  |  |  | 5/-7.869 | 3 | GLN 34, LYS 4, SER 31 |
|  |  | DLNC_ZINC04237100 | 1/-8.75 | 0 | No interaction |
|  |  |  | 2/-8.566 | 0 | No interaction |
|  |  |  | 3/-8.134 | 0 | No interaction |
|  |  |  | 4/-8.088 | 0 | No interaction |
|  |  |  | 5/-8.047 | 0 | No interaction |
|  |  | DLNC_ZINC04237101 | 1/-8.937 | 0 | No interaction |
|  |  |  | 2/-8.243 | 0 | No interaction |
|  |  |  | 3/-8.143 | 0 | No interaction |
|  |  |  | 4/-8.123 | 0 | No interaction |
|  |  |  | 5/-8.108 | 0 | No interaction |
|  |  | DLNC_ZINC04258896 | 1/-8.808 | 1 | PHE 80 |
|  |  |  | 2/-8.502 | 0 | No interaction |
|  |  |  | 3/-8.475 | 0 | No interaction |
|  |  |  | 4/-8.344 | 1 | LYS 4 |
|  |  |  | 5/-8.334 | 0 | No interaction |
|  |  | DLNC_ZINC04277699 | 1/-8.692 | 0 | No interaction |
|  |  |  | 2/-8.316 | 0 | No interaction |
|  |  |  | 3/-8.232 | 0 | No interaction |
|  |  |  | 4/-7.714 | 0 | No interaction |
|  |  |  | 5/-7.686 | 0 | No interaction |
|  |  | DLNC_ZINC04277705 | 1/-8.835 | 0 | No interaction |
|  |  |  | 2/-8.111 | 0 | No interaction |
|  |  |  | 3/-7.866 | 0 | No interaction |
|  |  |  | 4/-7.848 | 1 | PHE 80 |
|  |  |  | 5/-7.616 | 0 | No interaction |
|  |  | DLNC_ZINC05415069 | 1/-8.654 | 0 | No interaction |
|  |  |  | 2/-8.531 | 0 | No interaction |
|  |  |  | 3/-8.153 | 0 | No interaction |
|  |  |  | 4/-8.058 | 0 | No interaction |
|  |  |  | 5/-7.998 | 0 | No interaction |
|  |  | DLNC_ZINC05415074 | 1/-8.91 | 0 | No interaction |
|  |  |  | 2-8.328 | 0 | No interaction |
|  |  |  | 3/-7.85 | 0 | No interaction |
|  |  |  | 4/-7.778 | 0 | No interaction |
|  |  |  | 5/-7.743 | 0 | No interaction |
|  |  | DLNC_ZINC05415832 | 1/-8.837 | 0 | No interaction |
|  |  |  | 2/-8.737 | 0 | No interaction |
|  |  |  | 3/-8.457 | 0 | No interaction |
|  |  |  | 4/-8.334 | 0 | No interaction |
|  |  |  | 5/-8.275 | 0 | No interaction |
|  |  | DLNC_ZINC08300268 | 1/-8.644 | 0 | No interaction |
|  |  |  | 2/-8.519 | 0 | No interaction |
|  |  |  | 3/-8.374 | 0 | No interaction |
|  |  |  | 4/-8.293 | 0 | No interaction |
|  |  |  | 5/-8.264 | 0 | No interaction |
| WP_002295134.1 | sugar-binding transcriptional regulator | DLNC_ZINC04235906 | 1/-9.171 | 0 | No interaction |
|  |  |  | 2/-8.406 | 0 | No interaction |
|  |  |  | 3/-8.146 | 1 | ARG 137 |
|  |  |  | 4/-8.116 | 0 | No interaction |
|  |  |  | 5/-8.04 | 0 | No interaction |
|  |  | DLNC_ZINC04237089 | 1/-9.211 | 1 | ARG 109 |
|  |  |  | 2/-9.074 | 0 | No interaction |
|  |  |  | 3/-8.496 | 0 | No interaction |
|  |  |  | 4/-8.41 | 0 | No interaction |
|  |  |  | 5/-8.152 | 1 | LEU 247 |
|  |  | DLNC_ZINC04259022 | 1/-9.303 | 4 | GLU 98, GLU 98, GLU 98, ASN 86 |
|  |  |  | 2/-7.662 | 0 | No interaction |
|  |  |  | 3/-7.358 | 0 | No interaction |
|  |  |  | 4/-7.314 | 2 | TYR 217, GLU 293 |
|  |  |  | 5/-7.098 | 0 | No interaction |
|  |  | DLNC_ZINC04259094 | 1/-9.12 | 4 | VAL 112, GLU 98, GLU 133, GLU 133 |
|  |  |  | 2/-8.49 | 1 | ARG 109 |
|  |  |  | 3/-8.245 | 1 | ASN 86 |
|  |  |  | 4/-7.816 | 0 | No interaction |
|  |  |  | 5/-7.79 | 2 | ARG 109, HIS 95 |
|  |  | DLNC_ZINC05414523 | 1/-9.168 | 0 | No interaction |
|  |  |  | 2/-7.526 | 0 | No interaction |
|  |  |  | 3/-6.84 | 1 | GLU 85 |
|  |  |  | 4/-6.837 | 0 | No interaction |
|  |  |  | 5/-6.748 | 0 | No interaction |
|  |  | DLNC_ZINC05434233 | 1/-9.21 | 1 | GLU 133 |
|  |  |  | 2/-7.614 | 0 | No interaction |
|  |  |  | 3/-7.542 | 0 | No interaction |
|  |  |  | 4/-7.223 | 2 | CYS 110, CYS 110 |
|  |  |  | 5/-7.007 | 2 | ARG 245, SER 311 |
|  |  | DLNC_ZINC08297457 | 1/-9.216 | 0 | No interaction |
|  |  |  | 2/E-8.85 | 0 | No interaction |
|  |  |  | 3/-7.561 | 0 | No interaction |
|  |  |  | 4/-7.126 | 0 | No interaction |
|  |  |  | 5/-7.104 | 0 | No interaction |
|  |  | DLNC_ZINC08297458 | 1/-9.546 | 0 | No interaction |
|  |  |  | 2/-7.917 | 0 | No interaction |
|  |  |  | 3/-7.345 | 0 | No interaction |
|  |  |  | 4/-7.276 | 0 | No interaction |
|  |  |  | 5/-7.195 | 0 | No interaction |
|  |  | DLNC_ZINC08635277 | 1/-9.878 | 0 | No interaction |
|  |  |  | 2/-9.46 | 0 | No interaction |
|  |  |  | 3/-9.36 | 0 | No interaction |
|  |  |  | 4/-9.127 | 0 | No interaction |
|  |  |  | 5/-8.332 | 0 | No interaction |
|  |  | DLNC_ZINC20503278 | 1/-9.571 | 1 | ARG 109 |
|  |  |  | 2/-7.992 | 0 | No interaction |
|  |  |  | 3/-7.758 | 1 | THR 152 |
|  |  |  | 4/-7.548 | 2 | SER 117, THR 152 |
|  |  |  | 5/-7.488 | 0 | No interaction |

The best dockings interactions of each drug target are highlighted in yellow.

**Figure S1: Ramachandran's plot**

**
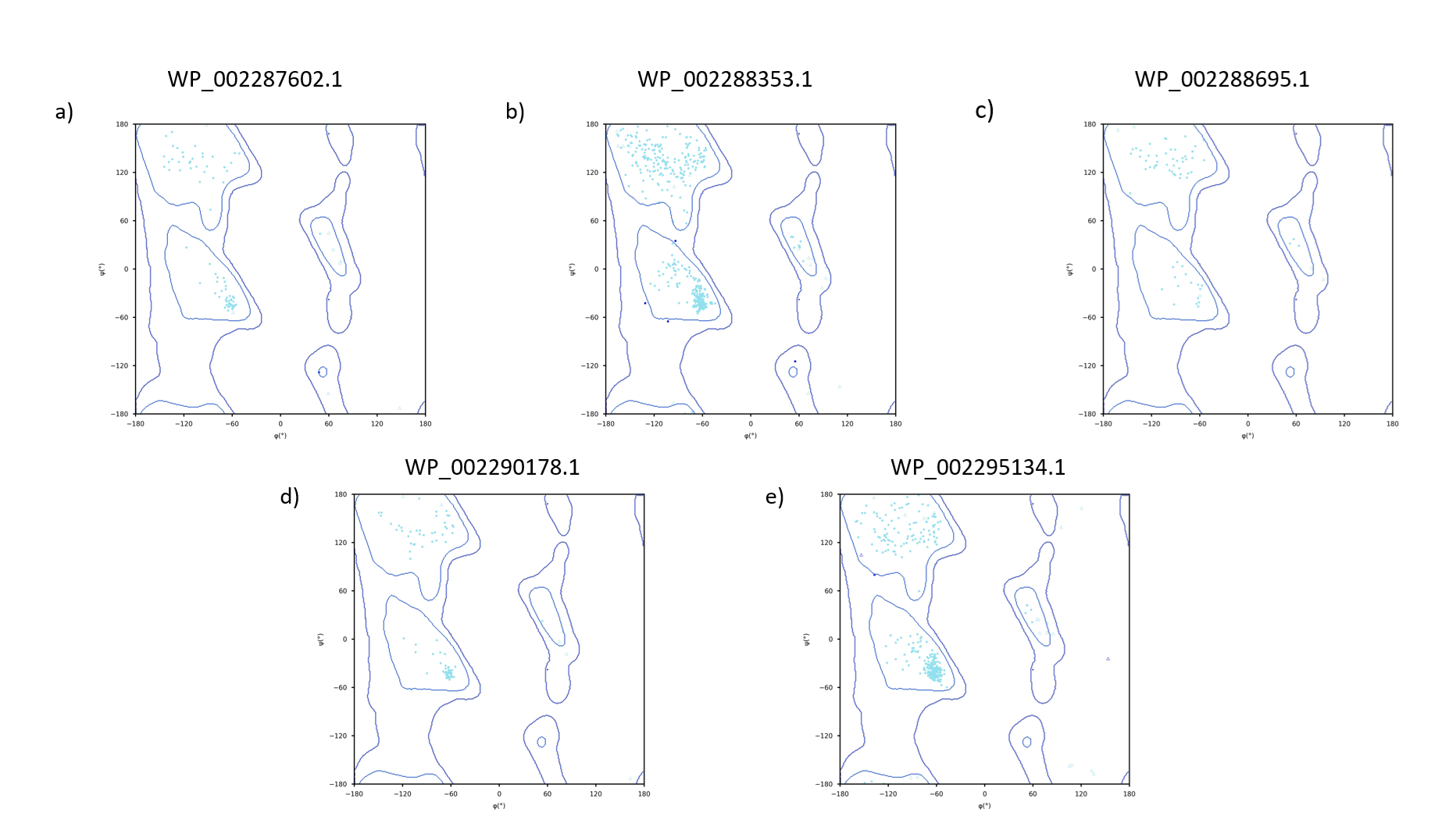
**

Each graph represents the Ramachandran plot for each protein: WP_002287602.1 - A; WP_002288353.1 - B; WP_002288695.1 - C; WP_002290178.1 - D; WP_002295134.1 - E.
